# Noble metal-modified faceted anatase titania photocatalysts: Octahedron versus decahedron

**DOI:** 10.1016/j.apcatb.2018.06.027

**Published:** 2018-12-05

**Authors:** Zhishun Wei, Marcin Janczarek, Maya Endo, Kunlei Wang, Armandas Balčytis, Akio Nitta, Maria G. Méndez-Medrano, Christophe Colbeau-Justin, Saulius Juodkazis, Bunsho Ohtani, Ewa Kowalska

**Affiliations:** a Hubei Provincial Key Laboratory of Green Materials for Light Industry, Hubei University of Technology, Wuhan, 430068, China; b Institute for Catalysis, Hokkaido University, N21, W8, 001-0021, Sapporo, Japan; c Department of Chemical Technology, Gdansk University of Technology, G. Narutowicza 11/12, 80-233, Gdansk, Poland; d Center for Micro-Photonics, Swinburne University of Technology, John St., Hawthorn, 3122 Vic, Australia; e Laboratory of Physical Chemistry, UMR 8000, University of Paris-Saclay, 91405, Orsay, France

**Keywords:** Noble metal, Faceted anatase titania, Octahedral anatase particle, Decahedral anatase particle, Plasmonic photocatalysis, 3D-FDTD simulations

## Abstract

Octahedral anatase particles (OAP, with eight equivalent {101} facets) and decahedral anatase particles (DAP, with two additional {001} facets) were modified with nanoparticles of noble metals (Au, Ag, Cu). The titania morphology, expressed by the presence of different arrangements of exposed crystal facets, played a key role in the photocatalytic properties of metal-modified faceted titania. In the UV/vis systems, two-faceted configuration of DAP was more favorable for the reaction efficiency than single-faceted OAP because of an efficient charge separation described by the transfer of electrons to {101} facets and holes to {001} facets. Time-resolved microwave conductivity (TRMC) and reversed double-beam photoacoustic spectroscopy (RDB-PAS) confirmed that distribution of electron traps (ET) and mobility of electrons were key-factors of photocatalytic activity. In contrast, metal-modified OAP samples had higher photocatalytic activity than metal-modified DAP and metal-modified commercial titania samples under visible light irradiation. This indicates that the presence of single type of facets ({101}) is favorable for efficient electron transfer via shallow ET, whereas intrinsic properties of DAP result in fast charge carriers’ recombination when gold is deposited on {101} facets (migration of “hot” electrons: Au→{101}→Au).

## Highlights

• Two-faceted (DAP) ― more favorable configuration than single-faceted (OAP) under UV irradiation.
• Bare and modified DAP ― efficient charge carriers’ separation under UV irradiation.
• Noble metal-modified OAP ― higher activity than respective DAP under vis irradiation.
• “Hot” (plasmonic) electron transfer via shallow electron traps in OAP.
• Localization of noble metal on DAP – key factor of vis activity.

## Graphical abstract

**Figure:**
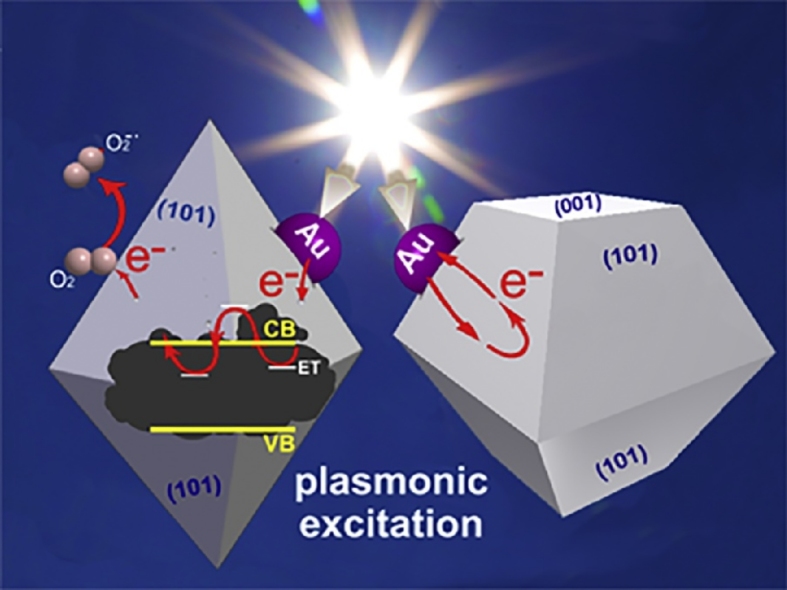

## 1 Introduction

Fossil energy exhaustion and environmental pollution are major challenges to sustain development of human society. Therefore, renewable technologies for environmental remediation and energy conversion, such as utilization of solar energy by semiconductor photocatalysis, are highly desired. Titanium(IV) oxide (titania, TiO_2_) has been one of the most widely studied semiconductor photocatalysts since Fujishima and Honda report in 1972 on photoelectrochemical water splitting using a titania electrode [1]. Titania is cheap, non-toxic (except toxicity of nanomaterials [2]), highly active (both for oxidation and reduction reactions of chemical compounds and microorganisms [3]), stable, and thus with a great potential for commercial applications [[4], [5], [6], [7]]. For example, titania has already been used for environmental purification (water [8] and air [9] treatment), renewable energy (water splitting with hydrogen generation [10] and/or conversion of CO_2_ to hydrocarbons [11], solar cells [12,13]) and self-cleaning surfaces [14]. However, two limitations of TiO_2_-photocatalysis should be overcome, i.e., (i) recombination of charge carriers (photogenerated electrons (e^−^) with holes (h^+^)), and (ii) poor overlap of the solar spectrum with the absorption spectrum of titania (absorption edge at ca. 385–410 nm due to wide bandgap of ca. 3.0–3.2 eV depending on crystalline form). Various methods of titania modification have been proposed to improve its photocatalytic performance, such as doping (with cations [[15], [16], [17]], anions [[18], [19], [20]] or self-doping [[21], [22], [23]]) [24], surface modification [[25], [26], [27]] and coupling with other materials (heterojunctions [[28], [29], [30]]). Recently, nano-architecture has gained large interest as efficient method allowing to design nanomaterials with demanded properties [[31], [32], [33]]. For example, preparation of highly crystalline polyhedral TiO_2_ particles with crystal facets in definite orientations has been reported as a promising strategy to retard charge carriers’ recombination [[33], [34], [35]]. To obtain visible-light-responsive photocatalysts, various compounds have been used as surface modifiers of titania, e.g., metals (in the form of metallic nanoparticles (zero-valent metal [27] or chemical compounds [36]), nonmetals (adsorbed anions [37] or chemical compounds [38]) and organic compounds (colorless [39] and color [40], such as dyes, used also in dye sensitized solar cells, DSSCs [12]).

Noble metals (NMs: Pt, Au, Ag, Ir, Pd) in the form of either adsorbed complexes [41] or metallic deposits [42] have been extensively studied for more than forty years as a pool for UV-photogenerated electrons retarding charge carriers’ recombination [[43], [44], [45]]. Very recently another property of NMs, i.e., visible light (vis) absorption due to plasmon resonance, has been used for activation of wide-bandgap semiconductors toward vis, i.e., mainly titania, but also other materials, such as CeO_2_ [46], Fe_2_O_3_ [47], ZnO [48] and KNbO_3_ [49]. Although plasmonic properties of NMs were observed and explained many years ago, and commercially used in various fields (e.g., SERS, medicine, optical data storage), the examination of their use for photocatalysis started ca. decade ago. Despite the novelty of plasmonic photocatalysis, a large number of studies have already been performed to improve photocatalytic activity and stability as well as to clarify the mechanism under vis; a few reviews on plasmonic photocatalysis have been also published [[50], [51], [52], [53]]. Application of localized surface plasmon resonance (LSPR) to photocatalysis started at the beginning of this century, but at this time plasmonic features were only used for characterization of gold NPs deposited on titania, i.e., their formation, properties (size/shape) and stability (under UV irradiation) [54]. Finally, gold-modified titania was used as a vis-responsive photocatalyst for photocurrent generation [55] and decomposition of organic compounds, such as methyl *tert*-butyl ether [56] and 2-propanol [57]. The first reports directly proving the responsibility of LSPR for vis activity were presented by action spectrum (AS) analyses [55,57], where AS resembled respective absorption spectra. Three main mechanisms have been proposed for plasmonic photocatalysis (under vis excitation):

1) Charge transfer (mainly electron transfer),
2) Energy transfer,
3) Plasmonic heating (thermal activation).
  1 In general, the mechanism of decomposition of organic compounds (OCs) on plasmonic photocatalyst under vis irradiation is similar to that of sensitizer activation, and thus NM is also called “plasmonic photosensitizer”. In brief, incident photons are absorbed by NMNPs through their LSPR excitation, and then an electron (“hot electron”) is transferred from NM into the CB of semiconductor. Then, the electron reduces molecular oxygen adsorbed on the semiconductor surface and the resultant electron-deficient NMNP can oxidize OCs to recover its original metallic state. There are plenty of reports indirectly or directly proving electron transfer mechanism, which involve different types of experiments, e.g., (i) femtosecond transient absorption spectroscopy with an IR probe of interband absorption of electrons injected from gold nanodots into CB of titania [58], (ii) shift of electrode potential (negative or positive photopotential) and generation of anodic or cathodic photocurrent depending on electrode configuration: ITO/TiO_2_/Au or ITO/Au/TiO_2_, respectively [59,60], (iii) EPR study resulting in detection of different species under irradiation with UV and vis [61,62].
  2 Energy transfer between two compounds can take place when they have closely matched energy levels, which is not expected for Au/TiO_2_ since plasmon energy of gold NPs (LSPR of ca. 2.2 eV) is much lower than bandgap of titania (ca. 3–3.2 eV). Therefore, the first studies suggesting energy transfer (plasmon resonance energy transfer, PRET) from gold NPs to titania were done for pre-modified titania (in order to make it able to absorb vis, thus being active under vis irradiation). For example, energy transfer was shown for photocurrent generation on 5-nm gold NPs on titania film pre-modified with nitrogen and fluorine [63]. Other pre-modified titania, e.g., with nitrogen [64], and narrow-bandgap semiconductors possessing activity under vis irradiation like CuWO_4_ (2.0–2.5 eV) [65], showed an enhancement of photocatalytic activity after addition of gold NPs, which was attributed to the PRET mechanism. Interestingly, titania with crystalline defects [66] and amorphous titania with disorders resulting in localized states inside bandgap (i.e., electron traps, ET) [67] were also proposed as good materials for PRET due to energy matching of these states and LSPR of gold.
  3 The first report suggesting plasmonic heating was published by Chen et al. in 2008, where it was presented that plasmonically heated gold NPs could activate organic molecules to induce their oxidation [68]. Although plasmonic heating was proposed as the main mechanism for some studies, there are plenty of reports rejecting this mechanism, mainly due to inactivity of gold-modified insulators or unsupported gold NPs in comparison to highly active gold-modified semiconductors. For example, plasmonic heating was not considered as the main mechanism in the case of: (i) water splitting [63], (ii) photocurrent generation [69], (iii) hydrogen dissociation [70]. Studies on activation energy also excluded plasmonic heating in the case of decomposition of OCs [71] and photocurrent generation [72].

Various structures of plasmonic photocatalysts have been proposed, which differ significantly in physicochemical properties (adsorption properties of the reagents, light absorption properties, and resultant activities). Therefore, it is not surprising that different photocatalytic mechanisms have been proposed for different nanostructures. Moreover, several mechanisms may be involved in the (photo)catalytic reaction simultaneously. Gold and silver are mainly used for plasmonic photocatalysis, but also other NMs have already been applied, such as palladium [73], platinum [74] and copper [75].

Although, desirable absorption properties of plasmonic photocatalysts can be easily obtained by preparation of NPs of different sizes and shapes, their photocatalytic activities under vis irradiation are still low and should be improved for commercial application. Therefore, various methods have been applied to enhance activity and stability of plasmonic photocatalysis, e.g., by extension of photoabsorption range (light harvesting), i.e., (i) preparation of NPs of various sizes and shapes [76], (ii) composed of two noble metals (e.g., Au-Ag, Ag-Cu, Ag-Pt, Au-Pd, Ag-Pd) [[77], [78], [79], [80], [81]], or (iii) combined with homogeneous photocatalysts (e.g., Ru complexes) [82,83]. Although, resultant photocatalysts have shown better photoabsorption properties, in many cases a decrease in vis photocatalytic activity has been observed since second modifier works as a recombination center [[82], [83], [84], [85]]. Recently, another method of activity enhancement has been proposed, i.e., nano-architecture design of plasmonic photocatalysts, such as deposition of NMNPs in/on the structure of aerogels [86], mesocrystals [87] and facetted titania.

**Fig. 1:**
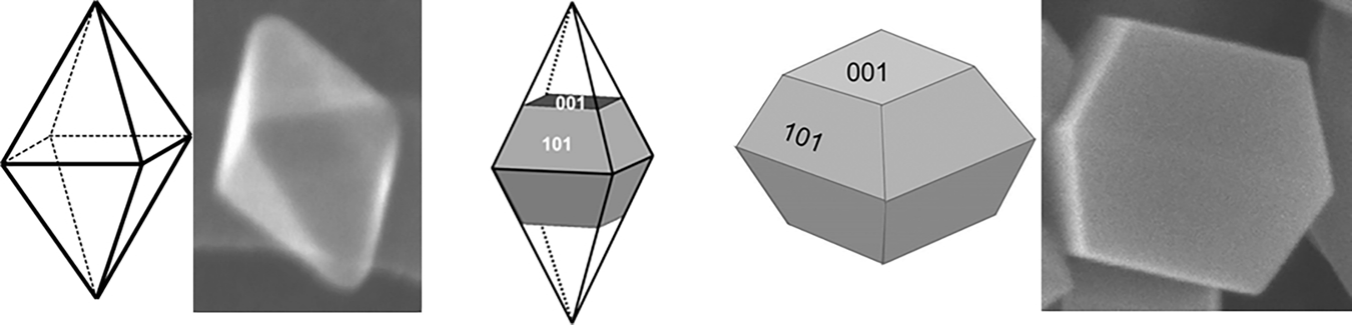
Schematic drawings with respective SEM images of OAP (left) and DAP (right), central drawing – DAP inside OAP (DAP formation during gas-phase method).

To obtain highly active plasmonic photocatalysts and to clarify the mechanism and key-factors of photocatalytic activity under vis irradiation, two types of faceted anatase nanoparticles (NPs) with well-controlled morphology (Fig. 1) and very high photocatalytic activity under UV irradiation (similar to that of P25) have been used in the present study, i.e., octahedral anatase particles (OAP) with eight exposed thermodynamically stable {101} facets [88] and decahedral anatase particles (DAP) with two types of crystal facets: {101} and {001} [89], as supports for NPs of NMs: gold, silver and copper. Although, some data for OAP and DAP have already been published (bare [[88], [89], [90]] and NM-modified faceted titania [[91], [92], [93]]), in this study, for the first time, NM-modified OAP and DAP have been comparatively analyzed to recognize how the different types of faceted anatase morphology can influence photocatalytic activity of samples modified by NMs in different reaction systems.

## 2 Experimental details

### 2.1 Preparation of photocatalysts

Octahedral anatase particles (OAP) with eight exposed thermodynamically stable {101} facets were prepared by an ultrasonication-hydrothermal reaction (US-HT) [88,94]. Decahedral anatase particles (DAP) with two types of crystal facets: {101} and {001}, were synthesized by a gas-phase method with TiCl_4_ as a titania precursor [89,95]. Schematic drawing showing OAP and DAP with respective SEM images are presented in Fig. 1. In brief, OAP was synthesized by HT (433 K, 6 h) reaction of potassium titanate nanowires (TNWs) preceded by 1-h US in order to homogenize TNWs’ suspension and to optimize the morphology of the final product (the content of octahedral particles) [88]. DAP was prepared from titanium(IV) chloride (TiCl_4_) and oxygen by rapid heating and quenching of gas reaction mixture (rapid quenching was necessary to avoid formation of thermodynamically stable OAP). TiCl_4_ was continuously fed to a vaporizer vessel heated at 453 K. Simultaneously, argon was passed through the vaporizer as a carrier gas for TiCl_4_ vapor. A mixture of two gases was introduced through a preaheating zone tube (473 K) to a quartz reaction tube located coaxially inside an infrared furnace. Oxygen was passed to the preaheated zone tube without the contact with Ar/TiCl_4_ stream, and further delivered to the quartz reaction tube. Its central part was wrapped with platinum foil and heated to 1373 K. DAP-containing powder was collected from the quartz reaction tube and the glass fiber filter in the downstream.

NM (gold, silver or copper; 2 wt% in respect to TiO_2_) was photodeposited on faceted titania (500 mg) in a sealed and deaerated (15-min argon pre-bubbling) tube containing methanol as a hole scavenger (25 mL, 50 vol%) and aqueous solution of NM (HAuCl_4_·4H_2_O, AgNO_3_, CuSO_4_·5H_2_O) under UV/vis irradiation. Details of photodeposition method were presented previously [96]. In brief, under UV irradiation of titania, photogenerated electrons reduce metal cations, and thus obtained metallic nanocluster/nanoparticles (NPs) are in situ deposited on titania. This method is very efficient due to the direct electronic contact between titania and metal NPs since metal cations are reduced by photogenerated electrons directly on the titania surface. Therefore, the linear evolution of hydrogen (methanol dehydrogenation during photodeposition) indicates that the reduction reaction is complete and all metal cations have been reduced and deposited on the support (The complete deposition of metals on titania by photodeposition method has been confirmed in previous studies by atomic absorption spectroscopy, e.g., for broad range of gold content (0.05–10 wt%) [76].).

The codes of samples were defined as DAP (anatase sample composed mainly of decahedral anatase particles), OAP (anatase sample composed mainly of octahedral anatase particles), Au/OAP, Ag/OAP and Cu/OAP (OAP sample with deposited gold, silver and copper, respectively), Au/DAP, Ag/DAP and Cu/DAP (DAP sample with desposited gold, silver and copper, respectively).

### 2.2 Characterization

Photoabsorption properties were analyzed by diffuse reflectance spectroscopy (DRS; JASCO V-670 equipped with a PIN-757 integrating sphere). Barium sulfate or bare faceted anatase samples (OAP/DAP) were used as a reference for DRS analysis. Crystalline properties of photocatalysts (crystalline phase content, crystallite size) were determined by X-ray powder diffraction (XRD; Rigaku intelligent XRD SmartLab with a Cu target). A crystallite size of anatase was estimated from the corrected width of (101) anatase diffraction peak using the Scherrer equation. The value of 0.891 was used as a constant in the Scherrer equation. Particle aspect ratio was calculated from the ratio of crystallite sizes estimated from the width of (004) and (101) diffraction peaks. Non-crystalline content, i.e., amorphous titania and water, was calculated by the internal standard method, in which highly crystalline nickel oxide (NiO) was used as the standard, by mixing titania (80 wt%) and NiO (20 wt%) samples.

Chemical composition of the surface (chemical state and content of elements) was analyzed by X-ray photoelectron spectroscopy (XPS; JEOL JPC-9010MC with MgKα X-ray). The morphology of samples (OAP, DAP, and size and distribution of NMNPs) was characterized by high resolution transmission electron microscopy (HR-TEM, JEOL JEM-2100 F) and scanning transmission electron microscopy (STEM) equipped with an energy-dispersive X-ray spectroscopy (STEM-EDS, HITACHI HD-2000). Summarized characterization of bare titania samples (OAP and DAP) is presented in Table 1.

**Table 1:** Structural properties of faceted anatase photocatalysts.

Name	Crystalline form^a^	Crystallinity^a^ (%)	Crystal size^a^ /nm	AR^a^	Particle size^b^ /nm	BET^c^ /m^2^ g^−1^	Morphology^d^ (%)	ETs^e^ /μmol g^−1^
OAP	anatase	78	17	1.71	32	124	64	34
DAP	anatase (97%) rutile (3%)	94	67	0.91	106	16	77	19

a Determined by XRD.

b Average size determined by SEM.

c Specific surface area estimated by Brunauer, Emmett and Teller method.

d Purity of morphology – content of faceted NPs in the product evaluated by SEM observation.

e Density of electron traps (ETs) estimated by RDB-PAS.

Charge-carrier dynamics was estimated by time-resolved microwave conductivity (TRMC) method. The incident microwave of 36.8 GHz was generated by a Gunn diode of the *K*α band, and UV laser pulses were obtained by the third harmonic of a 1064 nm Nd:YAG laser (10 Hz) with full width at half maximum of ca. 10 ns. Details of measurement and data processing have been reported elsewhere [97,98].

Energy-resolved density of electron traps was analyzed by reversed double-beam photoacoustic spectroscopy (RDB-PAS), according to the procedure described in previous report [99]. In brief, a particulate sample was set in a sample holder in a home-made aluminum PAS cell with a quartz window and a microphone, and methanol saturated argon gas passed through the cell for 30 min, and then the cell was closed. The upper side of the cell was irradiated by a 625-nm light-emitting diode beam (Luxeon LXHL-ND98), modulated at 80 Hz by a function generator (NF Corporation DF1906) as modulated light, and a monochromatic light beam from a xenon lamp (Spectral Products ASB-XE-175), equipped with a grating monochromator (Spectro Products CM110 1/8 m) as continuous light. The continuous light was scanned from 650 nm to 350 nm with a 5-nm step and the PA signal was acquired by a digital lock-in amplifier (NF Corporation LI5630). The obtained intensity of PA signal was converted to electron-trap density with a unit of μmol g^−1^ by comparison between total density of electron traps estimated by photochemical method [100] and saturated intensity of PA signal. Thus obtained spectrum was differentiated from the smaller energy side to obtain energy-resolved distribution of electron traps (ERDT) as a function of energy from top of valence band (VB). An original PA spectrum corresponding to photoabsorption spectrum was measured by a single light beam from the xenon lamp modulated by a light chopper at 80 Hz under nitrogen gas atmosphere. The conduction band (CB) bottom position was estimated from absorption edge of the PA spectrum.

The photocatalytic activity of prepared samples was evaluated in three reaction systems: (1) decomposition of acetic acid under UV/vis irradiation, (2) dehydrogenation of methanol under UV/vis irradiation, and (3) oxidation of 2-propanol under vis irradiation (λ > 420 nm: Xe lamp, water IR filter, cold mirror and cut-off filter Y45). For activity tests, 50 mg of photocatalyst was suspended in 5 ml of aqueous solution of (1) methanol (50 vol%), (2) acetic acid (5 vol%), and (3) 2-propanol (5 vol%). The 35-mL testing tubes were sealed with rubber septa, continuously stirred and irradiated in a thermostated water bath. Amounts of liberated (1) carbon dioxide in gas phase, (2) hydrogen in gas phase, and (3) acetone in liquid phase (after powder separation) were determined by gas chromatography (GC-TCD (1–2) and GC-FID (3)), details and experimental set-ups shown elsewhere [96,101].

Numerical simulations of light field enhancement was carried out by finite-difference time-domain (FDTD) method using the Lumerical software package. To calculate the cross sections of extinction, which is combined absorption and scattering, a standard procedure using total-field scattered-field light source was implemented. Calculations were carried out for the linearly polarized light using complex refractive index of titania (anatase) available in the database of Lumerical. Intensity of the plane wave illumination was set E^2^ = 1 providing direct image of light field enhancement at the vicinity of the contact between titania and gold nanoparticles. The effect of birefringence of anatase was tested, however, it was negligible due to the very small size of titania nanoparticles, which did not introduce measurable phase delays for different light field components. Calculations were carried out on Swinburne’s supercomputer.

## 3 Results and discussion

### 3.1 Photodeposition of metals on faceted titania

**Fig. 2:**
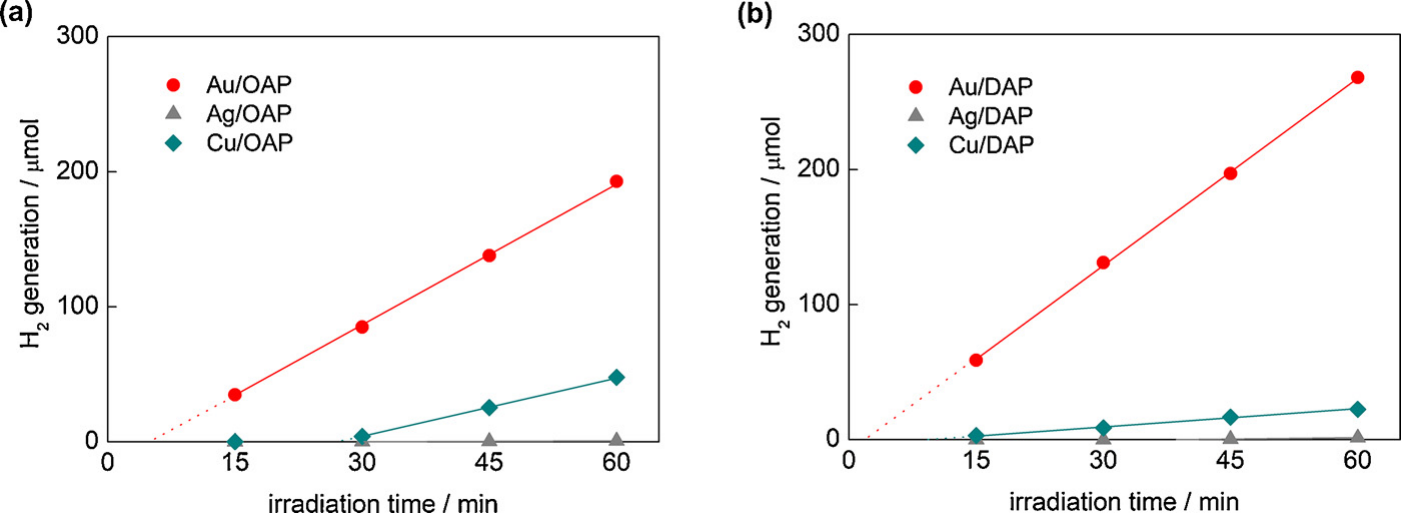
Hydrogen evolution during metal photodeposition on: (a) OAP and (b) DAP.

Faceted anatase particles (OAP/DAP) were modified with NM (Au, Ag or Cu; 2 wt%) NPs by photodeposition. Although the content of NMs has not been optimized (The “optimal content” depends on the properties of titania, and for vis activity for large titania particles (> 400 nm) an increase in gold content results in an activity increase (even up to 10 wt%), whereas for small titania particles (<30 nm) an increase in gold content results in a decrease in activity probably due to “inner filter” effect [76]), 2 wt% of NM has been selected for the study since usually it causes one of the highest activities under both UV and vis irradiation for various NMs and in different reaction systems, and is sufficient for samples’ characterization (XRD/XPS). During photodeposition the color of suspension changed from white to violet in the case of Au and Cu, and to brown for Ag confirming that zero-valent NPs of respective metals were deposited on titania (LSPR at 520–580 nm for spherical Au NPs [102] and Cu NPs [103], and at 410–430 nm for Ag NPs [104,105]). The photodeposition data showing hydrogen evolution rates and induction periods (during which NPs are formed) are presented in Fig. 2. The higher photocatalytic activity for hydrogen evolution (4.63 v. 3.39 μmol min^−1^) and the shorter induction period (intersection with the *x* axis in Fig. 2a,b) during Au deposition on DAP (ca. 2 min) than on OAP (ca. 5 min) confirm fast separation of charge carriers on DAP, due to co-existence of two kinds of facets, as reported previously [106]. Deposition of Cu needed longer irradiation duration, i.e., 10 min and 28 min for Cu/DAP and Cu/OAP, respectively, due to lower work function of Cu (4.61–4.67 eV) [107] than that of Au (5.1–5.45 eV) [108]. The longest induction period (as well as the lowest rates of hydrogen evolution) was observed for silver (>60 min) due to its lowest work function (4.14–4.46 eV) [109]. It should be mentioned that other factors, such as NMNPs’ size and hydrogen adsorption energy, could also influence the efficiency of hydrogen evolution. It seems that the influence of NMNPs’ sizes is not as crucial as other factors since silver NPs are smaller and more uniformly distributed than gold ones (Fig. 8, Fig. 10). However, hydrogen adsorption energy on gold, silver and copper [[110], [111], [112]] correlates well with photocatalytic activity of methanol dehydrogenation.

In contrast to gold-modified samples, shorter induction time for Cu/DAP than that for Cu/OAP does not correlate with photocatalytic activity (0.44 and 1.45 μmol min^−1^, respectively) indicating that the properties of Cu NPs (depending directly on titania support) could govern the activity. Indeed, finer Cu nano-clusters were uniformly deposited on OAP, whereas large Cu aggregates on DAP (Fig. 9), which is reasonable considering that copper cations would preferably adsorbed on {001} facet (more acidic than {101} [90]), which is only ca. 20% of whole DAP surface, resulting in NPs aggregation (Cu and Ag NPs are observed on both facets, whereas Au NPs (from AuCl_4_^−^ anion) only on {101}.). Moreover, it should be reminded that the reaction of hydrogen generation, i.e., proton reduction on the surface of co-catalyst (NMNPs), is caused by photogenerated electrons. Therefore, it is not surprising that in the case of DAP, co-deposition of Cu on two kinds of facets ({101} and {001}) results in lower activity of proton reduction since it is expected that photo-generated electrons would migrate to {101} facets, and photo-generated holes to {001} facets, and thus part of Cu-deposits (those on {001} facets) could be inactive.

### 3.2 Photoabsorption properties (DRS)

Although, surface modification of titania with metal NPs caused coloration of all samples due to plasmon resonance of NMs, only gold samples kept the same color after sample drying as that during photodeposition (violet). The color of other samples changed to violet-blue (Ag/DAP), light brown-violet (Ag/OAP), light grey-violet (Cu/DAP) and light green (Cu/OAP), since these less NMs are easily oxidized under aerobic conditions, and thus resultant photocatalysts possess both metallic and oxidized form of modifiers, regardless of the preparation method (e.g. zero-valent core and oxide shell) [77].

**Fig. 3:**
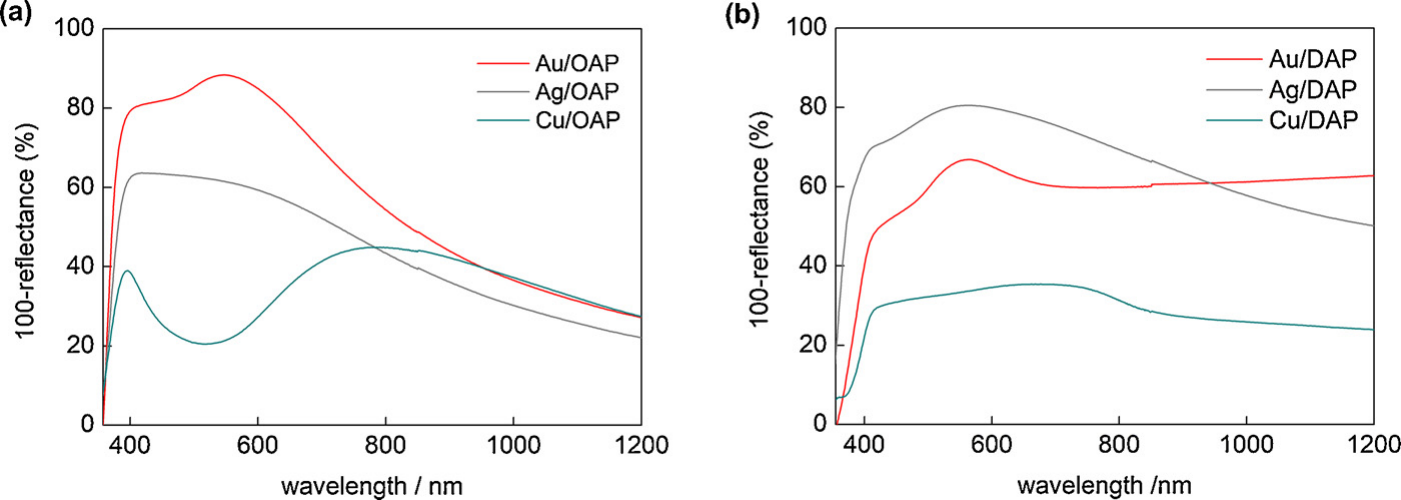
DRS spectra of metal-modified OAP (a) and DAP (b) samples taken with bare facetted titania (OAP and DAP, respectively) as reference.

**Fig. 4:**
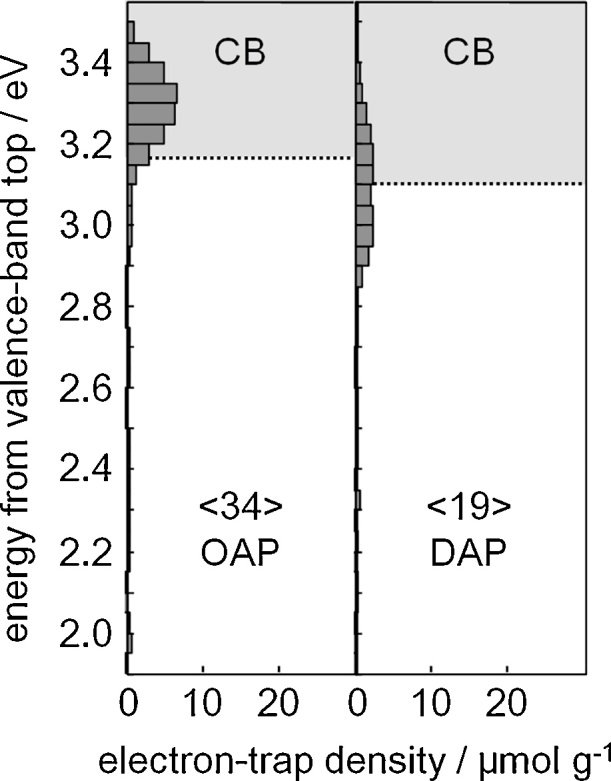
Energy-resolved distribution of ET for OAP and DAP.

DRS spectra of bare and modified faceted titania samples are shown in Figs. 3 and S1–S2. The intrinsic interband absorption of titania was observed at wavelengths shorter than 400 nm (E_g_ > 3 eV) for both titania samples (Fig. S1). However, DAP exhibited absorption tail till ca. 440 nm (ca. 2.8 eV), which could be explained as a result of the presence of: (i) impurity, (ii) Ti^3+^, and (iii) rutile phase. Although, DAP is very pure material, since only two chemical compounds (TiCl_4_ and O_2_) are used for its synthesis, a small amount of chlorine has been detected on its surface (less than 0.5 wt%, by XPS). The chlorine on DAP surface could be responsible for vis absorption, as was reported previously for Cl-contained TiO_2_ photocatalysts [113,114]. Although, the presence of Ti^3+^ was less possible since DAP was obtained in excess of oxygen in the system, Ti^3+^ was detected by XPS (Table 5) and RD-PAS (Fig. 4; electron traps (ETs) inside bandgap) methods. Therefore, it is proposed that, extreme thermal conditions in the oven, i.e., rapid heating to very high temperature (1373 K) and rapid quenching of reaction mixture (only ca. 1-s residence time of the reaction mixture in the oven) could result in the formation of both lattice defects and rutile phase. Indeed, DAP possesses small content of Ti^3+^ on the surface (2 mol% by XPS, Table 5) and rutile (3 wt%), as discussed in the next sections.

Both gold modified samples have characteristic plasmon peak (LSPR) with maximum at ca. 551 nm and 572 nm for Au/OAP and Au/DAP, respectively (Comparison of DRS spectra for single metal and different titanias is shown in Fig. S2). LSPR at longer wavelengths for Au/DAP than that for Au/OAP indicates that larger Au NPs (or/and with different than spherical shape) were formed on DAP than that on OAP. In addition, strong scattering observed at longer wavelengths than LSPR (λ > 800 nm) for Au/DAP confirms the presence of large gold deposits in this sample. Photoabsorption properties of silver-modified samples differ for Ag/DAP and Ag/OAP, as clearly shown (Fig. S2b) for spectra taken with bare titanias (DAP and OAP, respectively) as a reference (use of bare titania is necessary since the position of LSPR of spherical silver NPs is near interband absorption of titania). Although for both samples two LSPR peaks could be observed, i.e., at shorter (ca. 405 nm) and longer (ca. 505 nm) wavelengths, the intensity of both peaks differs. Thus, analogically to gold-modified samples, it may be concluded that Ag/DAP consists of larger Ag NPs with high polydispersity (more intense peak at longer wavelength), while smaller Ag NPs are obtained during photodeposition on OAP. This is not surprising since OAP possesses much smaller titania NPs than DAP, as shown in Table 1, and thus the presence of fine titania hinders the aggregation of metal NPs (less possible contact between two Ag NPs, similar as in the case of Au-modified samples). In the case of Cu, the color of samples and photoabsorption properties of faceted titania samples differ significantly (Fig. S2c). DRS spectrum of Cu/OAP with broad absorption peak in the range of 600–1000 nm indicates the presence of Cu(II), as reported for Cu-modified titania [77,92,115]. In contrast, photoabsorption of Cu/DAP with maximum at ca. 700 nm suggests Cu(I) [103,116] as the main Cu form in this sample. The co-existence of different forms of Cu (Cu(0), Cu_2_O, CuO and Cu_x_O (x = 1–2) [116,117]) is highly possible, especially in the case of Cu/DAP sample (due to intensive and broad absorption peak from 400 till 800 nm (confirmed by XPS analysis – discussed in the following section). For example, the interfacial charge transfer (IFCT) from the valence band of titania to the Cu_x_O clusters at 400–500 nm [117,118] and LSPR of zero-valent Cu at 550–570 nm [103] have been reported. It should be pointed that green color of Cu/OAP sample could also suggest plasmonic properties, as recently reported for Cu/TiO_2_ aerogels by DeSario et al [75]. It was proposed that ellipsoidal shape of 5-nm gold NPs resulted in significant bathochromic shift of Cu LSPR to wavelengths longer than 700 nm. Moreoever, the covering layer of Cu(I/II) oxides could also result in red-shift of LSPR (more than 100 nm), as was confirmed for copper nanoparticles covered with copper oxide layers with differen thicknesses [119].

### 3.3 Crystalline properties (XRD)

**Fig. 5:**
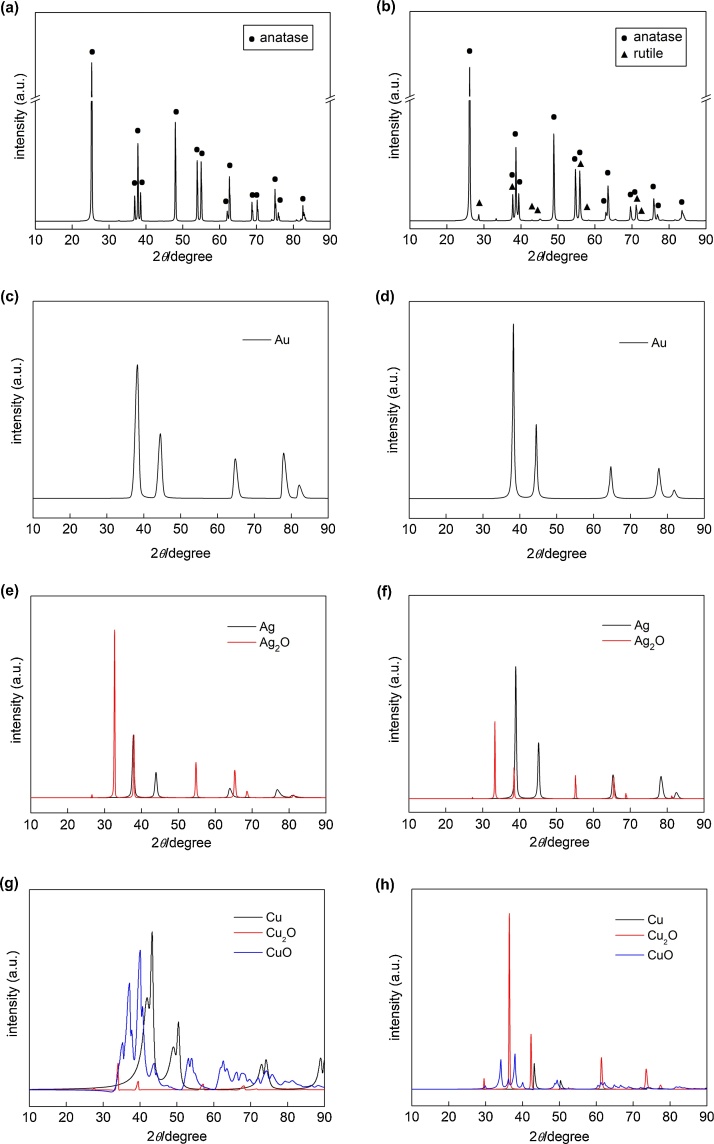
XRD patterns of (a) Ag/OAP with marked anatase peaks, (b) Ag/DAP with marked anatase and rutile peaks, (c–h) modified titania after subtraction of titania peaks: (c) Au/OAP, (d) Au/DAP, (e) Ag/OAP, (f) Ag/DAP, (g) Cu/OAP, (h) Cu/DAP.

The properties of bare titania photocatalysts (OAP and DAP) were summarized in Table 1. Both samples were mainly composed of anatase: 100% for OAP and 97% for DAP (Fig. 5a,b). However, crystallinity of DAP was better than that of OAP (94% v. 78%), as well as purity in morphology (content of faceted NPs in the product, i.e., 77% v. 64%). This is not surprising due to dynamic equilibrium between precipitation and dissolution of substrate during HT process (OAP preparation), which results in some wire-like structures and amorphous titania remaining in the final OAP product. On the other hand, DAP crystals are much larger than OAP crystals (ca. three times) resulting in much lower specific surface area (16 v. 124 m^2^g^−1^).

In the case of gold-modified samples, clear crystallographic peaks for metallic gold could be observed in the case of both samples (Fig. 5c,d). As expected based on DRS data (Figs. 3 and S2a), the crystal size of gold NPs was smaller in Au/OAP (5.7 nm) than that in Au/DAP (14.7 nm). It is proposed that small NPs of OAP hinder the aggregation of gold NPs (TiO_2_-Au-TiO_2_). Crystallographic data for silver-modified samples indicated similar composition of both samples (Fig. 5e,f), i.e., zero-valent silver and silver(I) oxide. However, in the case of DAP, larger content of silver(0) was observed (37.5% v. 60.1%). This is reasonable since larger NPs of silver were formed on DAP (Fig. 8 and Table 2). Considering that zero-valent silver deposits were formed first (anaerobic conditions) and afterwards their surfaces were oxidized in air resulting in formation of Ag(0) core-Ag_2_O shell, thus the larger metallic NPs were, the smaller was the content of oxide shell in the core-shell NP. In the case of Cu-modified samples, the ratio Cu:Cu_2_O:CuO was 0.84:0.02:1 and 0.18:0.8:1 for OAP and DAP, respectively. It should be reminded that NPs of zero-valent metal exist in crystalline form, whereas metal oxides could form amorphous structures, especially as a result of sluggish oxidation. Therefore, the content of Cu(0), estimated by XRD, could be higher than actual one. Moreover, it should be pointed that low content of metals (2 wt%) and small sizes of crystallites are hardly detected by XRD (thus, XPS analysis was also performed, and obtained data are presented in the next section). Despite some difficulties in enequivocal interpretation of XRD results, it is clear that Cu/OAP consists of significantly less cuprous oxide than Cu/DAP confirming DRS data. Summarized crystallographic data with other properties of NM-modified samples are shown in Table 2.

**Table 2:** Properties of NMs’ deposits and photoabsorption properties of metal-modified titania samples.

Titania	NM	Sample color	λ_max_^a^ (nm)	Predominant crystalline form^b^	Crystal size of zero-valent metal^b^ /nm	Crystal size of metal oxide^b^ /nm
OAP	Au	violet	544	Au	5.7	–
DAP		violet	563	Au	14.7	–
OAP	Ag	l. brown-violet	415	Ag_2_O	7.5	14.6 (Ag_2_O)
DAP		violet-blue	554	Ag	37.1	64.2 (Ag_2_O)
OAP	Cu	l. green	789	CuO	8.1	5.7 (CuO)
DAP		l. grey-violet	676	CuO	44.9	67.5 (CuO)

a maximum extinction from DRS.

b estimated from XRD.

### 3.4 Chemical properties of surface (XPS)

**Fig. 6:**
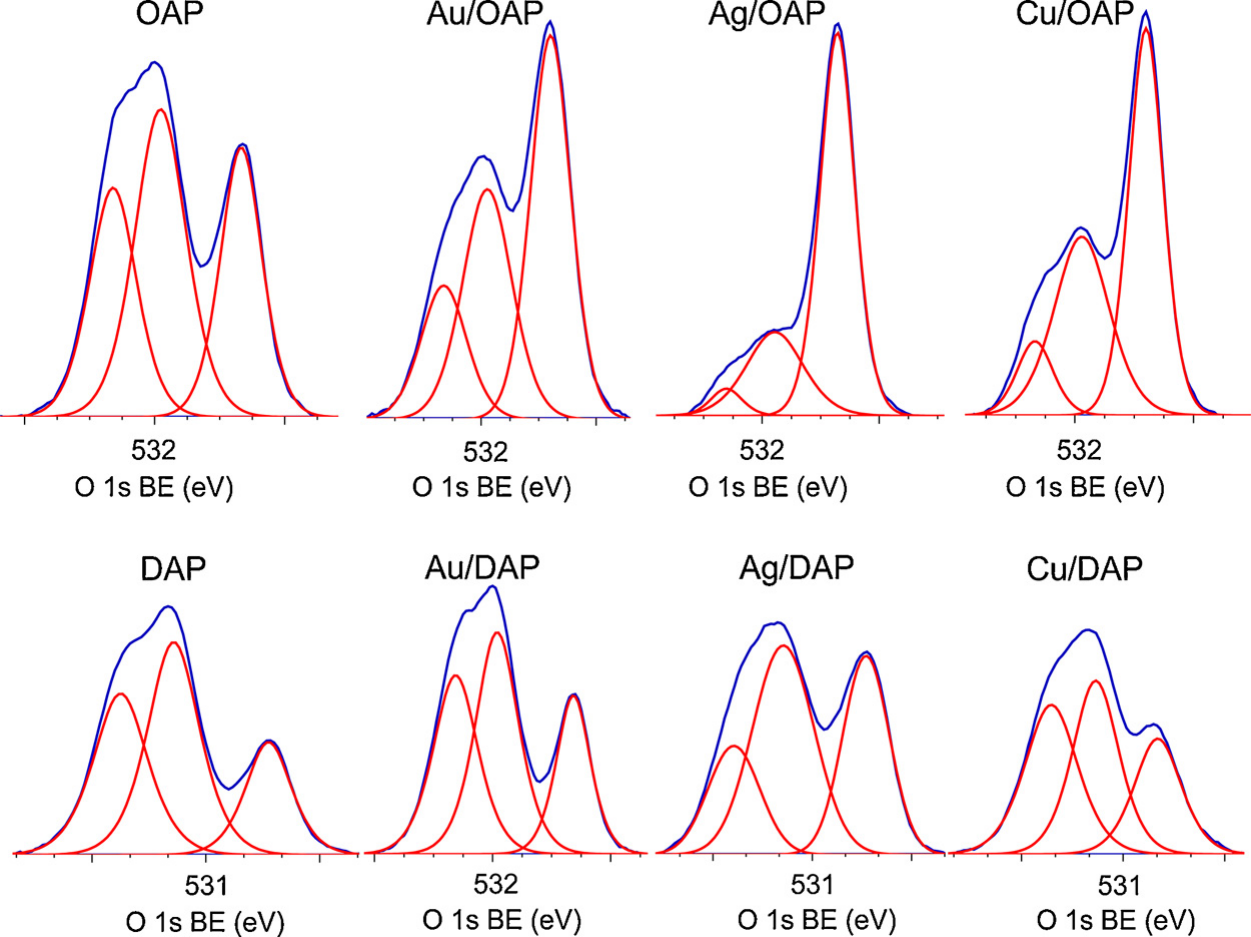
XPS results for O 1s for bare and metal-modified OAP (top line) and DAP (bottom line) samples.

**Fig. 7:**
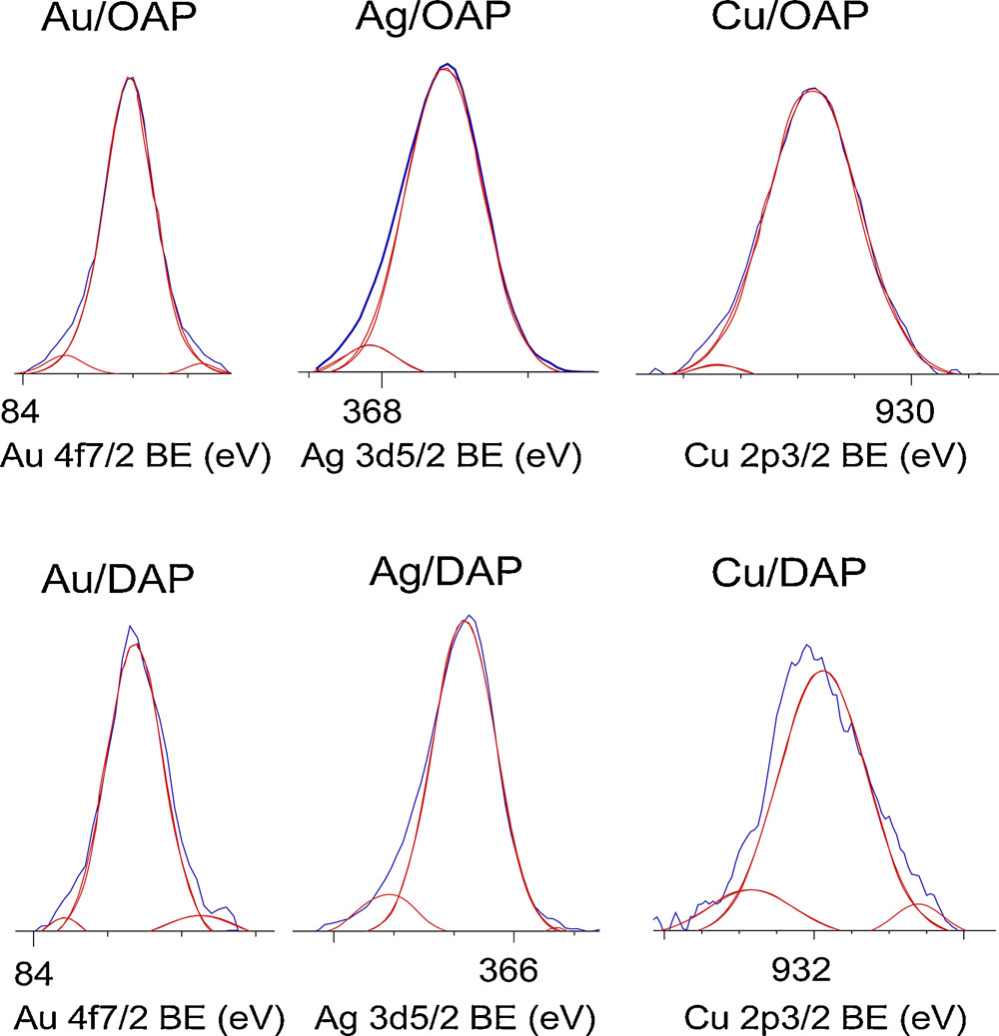
XPS results for Au 4f_7/2_, Ag 3d_5/2_ and Cu 2p_3/2_ for metal-modified OAP (top line) and DAP (bottom line) samples.

The chemical composition of samples was estimated by XPS analysis, and the obtained data are shown in Table 3, Table 4, Table 5 and Fig. 6, Fig. 7. The banding energies for titanium, oxygen, carbon, gold, silver and copper have been estimated after deconvolution in accordance to published reports on XPS analysis of titania samples [[120], [121], [122], [123], [124], [125]]. Narrow scan XPS spectra for O 1s, Au 4f_7/2_, Ag 3d_5/2_ and Cu 2p_3/2_ are shown in Fig. 6, Fig. 7. In the case of gold-modified titania samples, Au was predominantly in the zero-valent form both in Au/OAP and Au/DAP samples reaching 93.2% and 87.8%, respectively (see Table 4). Different situation was observed for silver and copper. Silver was recognized mainly as Ag^+^ (93.7% for Ag/OAP and 89.5% for Ag/DAP) and minority of zero-valent silver was also detected (6.3% and 10.0%, respectively). All forms of copper were found in Cu/DAP sample, and cuprous form was predominant in both faceted samples (82.0% for Cu/DAP and 97.8% for Cu/OAP). The 5.2% of Cu(0) was detected in Cu/DAP sample, but there was no zero-valent copper in Cu/OAP sample. It should be pointed out that XPS analysis gave information on the surface composition, and thus it was proposed that in both samples (also in Cu/OAP sample), metallic Cu core (based on XRD data) was covered with cuprous layer.

**Table 3:** Surface composition of bare and modified samples determined by XPS analysis of oxygen, titanium, carbon, metals for various samples.

Samples	Content (at. %)						Ratio		Metal content (wt. %)		
	Ti	O	C	Au	Ag	Cu	O/Ti	C/Ti	Au	Ag	Cu
OAP	5.3	27.8	66.9	–	–	–	5.2	12.6	–	–	–
Au/OAP	9.5	31.6	58.7	0.17	–	–	3.3	6.2	4.4	–	–
Ag/OAP	19.5	40.5	39.0	–	0.97	–	2.1	2.0	–	6.7	–
Cu/OAP	12.2	33.9	53.3	–	–	0.62	2.8	4.4	–	–	4.0
DAP	3.4	26.2	70.4	–	–	–	7.7	20.7	–	–	–
Au/DAP	4.0	26.6	69.4	0.06	–	–	6.7	17.4	3.7	–	–
Ag/DAP	6.5	27.9	64.9	–	0.76	–	4.3	10.0	–	15.8	–
Cu/DAP	3.6	26.2	69.6	–	–	0.59	7.3	19.3	–	–	13.0

**Table 4:** Fraction of oxidation states of Au, Ag and Cu from deconvolution of XPS peaks of Au 4f_7/2_, Cu 2p_3/2_ and Ag 3d_5/2_.

Samples	Valent state (%)			Valent state (%)			Valent state (%)		
	Au(δ+)	Au(0)	Au(δ-)	Ag^2+^	Ag^+^	Ag(0)	Cu^2+^	Cu^+^	Cu(0)
Au/OAP	4.7	93.2	2.1	–	–	–	–	–	–
Ag/OAP	–	–	–	0.0	93.7	6.3	–	–	–
Cu/OAP	–	–	–	–	–	–	2.2	97.8	0.0
Au/DAP	4.0	87.8	8.2	–	–	–	–	–	–
Ag/DAP	–	–	–	0.5	89.5	10.0	–	–	–
Cu/DAP	–	–	–	–	–	–	12.8	82.0	5.2

**Table 5:** Fraction of oxidation states of Ti, O and C from deconvolution of XPS peaks of Ti 2p_3/2_, O 1s and C 1s.

Samples	Ti 2p_3/2_ (%)		O 1s (%)			C 1s (%)		
	Ti^4+^	Ti^3+^	TiO_2_	Ti—OH^a^	Ti—OH^b^	C—C	C—OH	C=O
OAP	99.5	0.5	29.6	41.9	28.5	68.2	20.7	11.1
Au/OAP	97.5	2.5	46.9	34.5	18.6	73.5	16.8	9.7
Ag/OAP	97.3	2.7	70.3	25.2	4.5	82.5	9.2	8.3
Cu/OAP	97.9	2.1	51.6	38.0	10.4	69.7	20.6	9.7
DAP	98.0	2.0	20.8	44.2	35.0	71.1	17.5	11.4
Au/DAP	96.8	3.2	23.5	41.7	34.8	68.2	21.0	10.8
Ag/DAP	98.2	1.8	33.3	46.4	20.3	75.7	13.0	11.3
Cu/DAP	96.1	3.9	25.5	37.5	37.0	73.0	15.5	11.5

Ti—OH^a^: Ti—(OH)—Ti/Ti_2_O_3_/C=O, Ti—OH^b^: Ti—OH/ C—OH.

The ratio of oxygen to titanium (Table 3) exceeded stoichiometric one (2.0), reaching 5.2 and 7.7 for OAP and DAP, respectively, which has been often reported for titania samples. For example, enrichment of titania surface with oxygen was reported for samples prepared by the microemulsion method (O/Ti = 4.6) [85] and the laser ablation (O/Ti = 2.5) [126]. The larges O/Ti ratio for bare DAP is reasonable since decahedral particles have been synthesized under excess of oxygen (continuous flow) [89]. Deconvolution of oxygen peak into three peaks (Fig. 6) suggests the presence of three forms of oxygen, i.e., (i) oxygen in the crystal lattice of TiO_2_, (ii) C=O, Ti_2_O_3_ and OH groups bound to two titanium atoms, and (iii) hydroxyl groups bound to titanium or carbon (Ti—OH and C—OH) at ca. 529.5 eV, 531.8 eV and 533.3 eV, respectively [125]. Metals compete with oxygen for adsorption on the titania surface, as seen by much lower content of oxygen in metal-modified samples, especially in the case of fine crystals of OAP, i.e., a decrease in O/Ti ratio from 5.2 to 3.2, 2.1 and 2.7 after OAP modification with Au, Ag and Cu, respectively, and for DAP samples from 7.7 to 6.7, 4.3 and 7.3, respectively (Table 3).

Interestingly, the content of metals on the surface correlates with a decrease in O/Ti ratio, i.e., the lowest O/Ti ratios have been detected for silver-modified samples (2.1 for Ag/OAP and 4.3 for Ag/DAP) for which the largest content of metals are obtained, i.e., 6.7 wt% and 15.8 wt%, respectively. The smallest change in O/Ti ratio for Cu/DAP confirms that the surface of this sample contains copper almost only in the form of oxide. In the case of Cu/OAP, a decrease in O/Ti ratio results from the presence of zero-valent copper.

It is thought that metal NPs replaced mainly hydroxyl groups adsorbed on the surface of titania since significant increase in the content of lattice oxygen (TiO_2_) and a decrease in the content of hydroxyl oxygen (—OH) was observed after metal deposition, especially for OAP samples, as shown in Table 5. For example, OAP possesses 29.6% of oxygen in the form of TiO_2_ and similar amount (28.5%) in the form of surface hydroxyl groups (Ti—OH/C—OH), after modification with Ag the content of lattice oxygen increased to 70.3%, while the content of oxygen in the form of surface hydroxyl decreased to 4.5%. In contrast, modification of DAP with Au and Cu did not influence the surface composition of DAP significantly. Au NPs were primarily formed on {101} facets, and thus it could be concluded that in the case of decahedral sample, the hydroxyl groups might be mainly adsorbed on {001} facets. Therefore adsorbed gold on {101} facets does not compete with hydroxyl groups adsorbed on another facet.

Considering chemical state of titanium, both bare samples consist mainly Ti^4+^, and the content of Ti^3+^ reaches only ca. 0.5% and 2.0% for OAP and DAP, respectively (Table 5). This is reasonable since DAP is obtained at drastic changes of temperature, which could result in the formation of chemical defects. Interestingly, metal photodeposition resulted in an increase in the content of Ti^3+^ for all samples, except Ag/DAP. An increase in Ti^3+^ content is reasonable since the conditions during photodeposition are highly reductive (photogenerated electrons under anaerobic conditions in the presence of methanol as a hole scavenger). Slight decrease in the content of Ti^3+^ for Ag/DAP suggests that for those much larger particles than OAP, Ag was predominantly formed on lattice defects.

### 3.5 Morphology of photocatalysts (STEM)

**Fig. 8:**
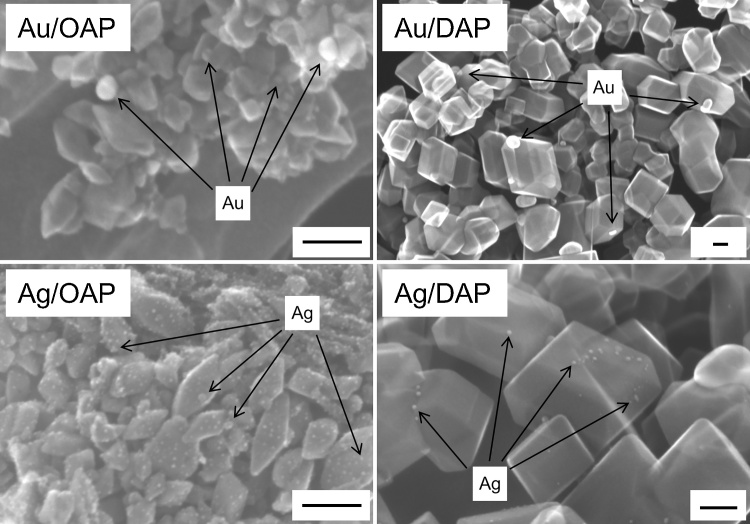
STEM (SE mode) images of (top) Au-modified titania: Au/OAP (left) and Au/DAP (right); (bottom) Ag-modified titania: Ag/OAP (left) and Ag/DAP (right). All scale bars correspond to 50 nm.

**Fig. 9:**
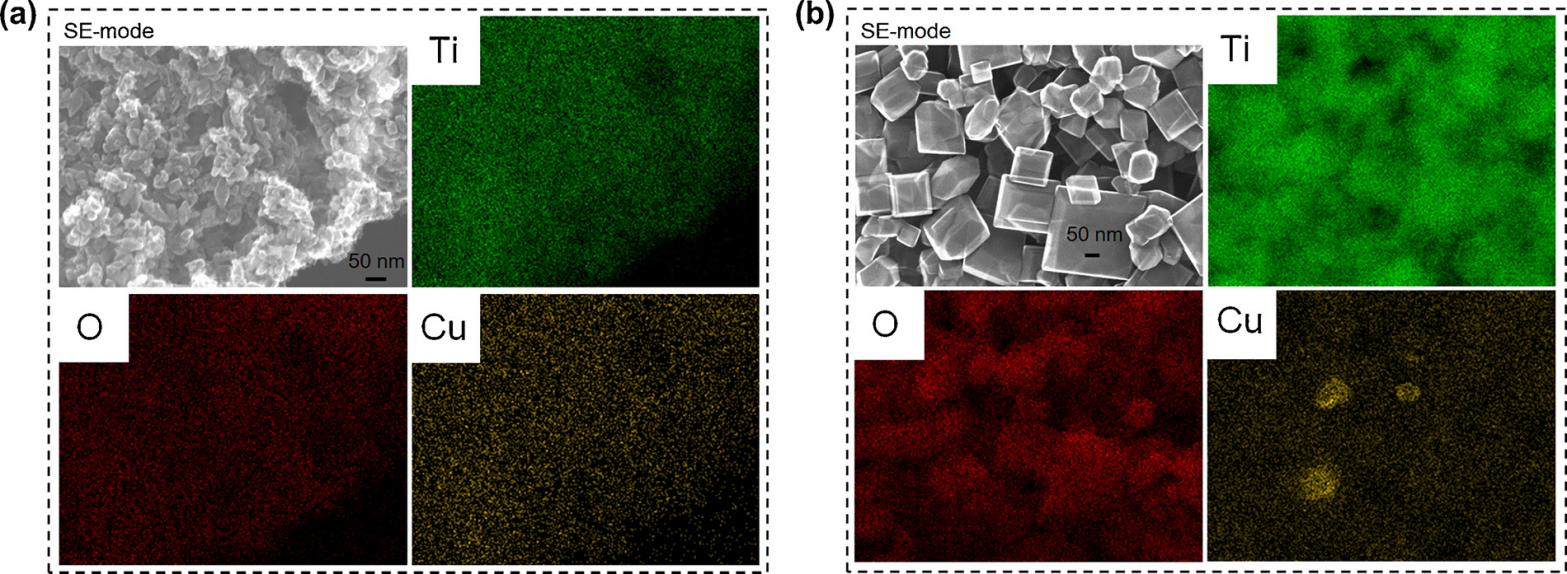
STEM images with respective EDS images of (a) Cu/OAP and (b) Cu/DAP. Mapping colors: Ti, green; Ag, red; Cu, yellow; SE-mode, scanning mode of STEM (For interpretation of the references to colour in this figure legend, the reader is referred to the web version of this article).

**Fig. 10:**
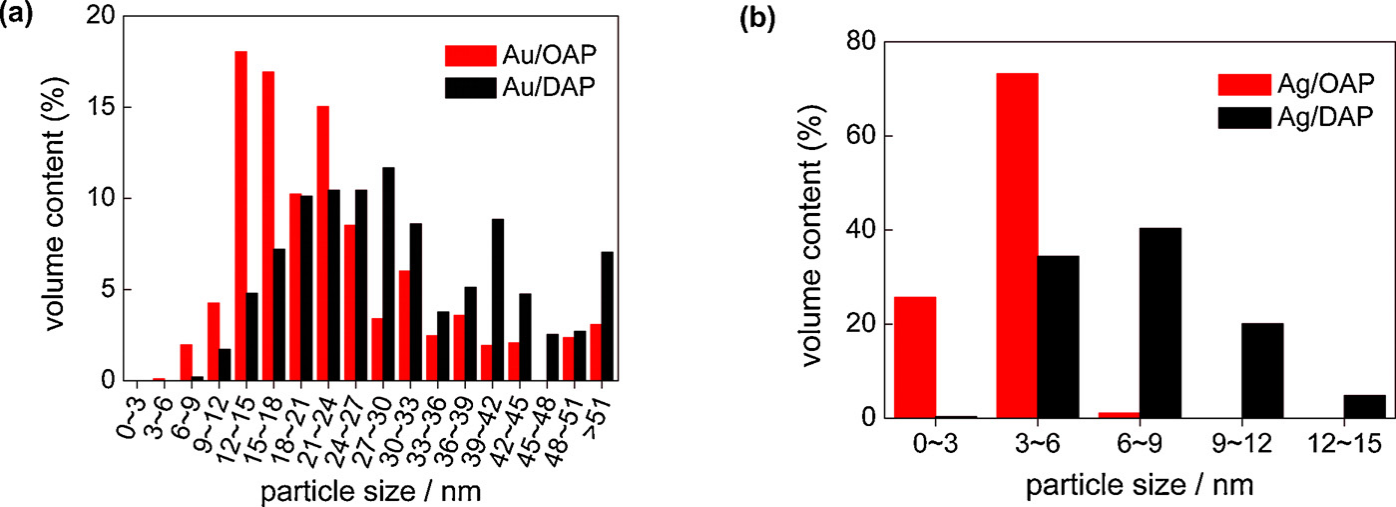
Particle size distribution of Au (a) and Ag (b).

Morphology of photocatalysts was studied by STEM and HR-TEM, and exemplary images are shown in Figs. 8 and S3–4. Au, Ag and Cu NPs were deposited on OAP and DAP surfaces. Cu NPs were not clearly visible in STEM images in SE mode, and thus the presence of copper NPs was confirmed by STEM images with EDS mapping (Fig. 9). In case of DAP samples, deposition of Au NPs occurred mainly on {101} facets, however, Ag NPs and Cu NPs were deposited on both facets (Selectivity of NMNPs deposition on DAP depends on many factors, such as NMNPs content, deposition conditions, precursor kind (cationic/anionic); Usually noble metals are mainly deposited on {101} facets due to direct reduction by photogenerated electrons on this facet. However, precursor of NM is also important as well as initial charge of DAP surfaces ({101}>{001} [90]) and thus chloroauric anions are easily adsorbed on more positive {101} surface, whereas copper and silver cations on more negatively charged {001} surface.). The presence of larger Au NPs on DAP than OAP, as earlier suggested by XRD and DRS analyses, was confirmed by STEM observation (Fig. 8, Fig. 10a). Similar to gold-modified samples, Ag/DAP and Cu/DAP consist of larger and more polydispersed NPs than that of modified OAP samples (Fig. 8, Fig. 9, Fig. 10b) since small size of OAP (Table 1) hinders aggregation of metal NPs.

### 3.6 Photocatalytic activity and the property-activity correlations

**Fig. 11:**
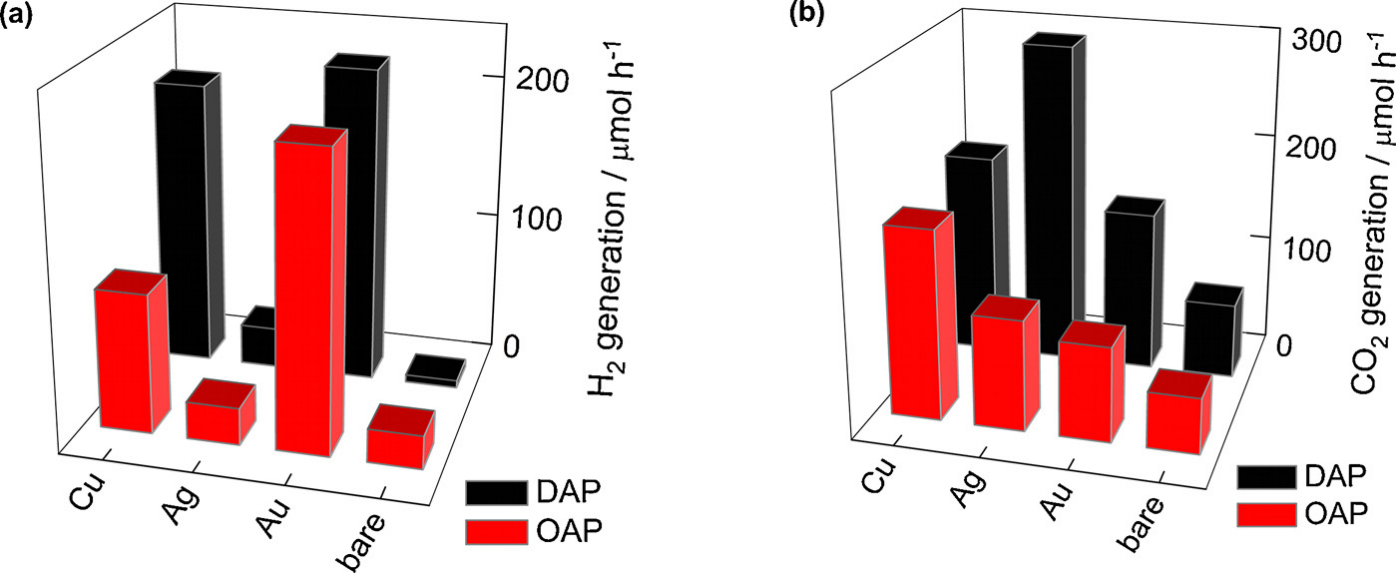
UV–vis-photocatalytic activity for methanol dehydrogenation (a) and decomposition of acetic acid (b) on bare and metal-modified OAP and DAP.

Although bare titania samples are usually inactive for hydrogen evolution, OAP possesses some activity exceeding DAP ca. 5 times (Figs. 11a and S5). It is proposed that shallow ET with high energy (inside conduction band (Fig. 4); detected by RDB-PAS [99]) are responsible for this activity. The highest photocatalytic activities of Au-modified samples agree with the highest work function of Au (5.1–5.45 eV vs. 4.61–4.67 eV (Cu) and 4.14–4.46 eV (Ag)) [109,127,128] and the smallest overvoltage for hydrogen evolutions. DAP samples after modification with Au and Ag showed slightly higher photocatalytic activity than that of respective OAP samples, and significantly higher photocatalytic activity after modification with Cu (which was unexpected considering activities during photodeposition, where Cu/DAP was ca. 4 and 10 times less active than Cu/OAP and Au/DAP, respectively). Considering differences in oxidation state of Cu (during and after photodeposition), it is concluded that efficient hydrogen evolution occurs on Cu-Cu_2_O-TiO_2_ heterojunction (as suggested by reference experiments for physical mixtures of copper oxides with titania, Fig. S8; Further investigations on this interesting system (Cu-Cu_2_O-TiO_2_) for other titania samples are presently under extensive study).

**Fig. 12:**
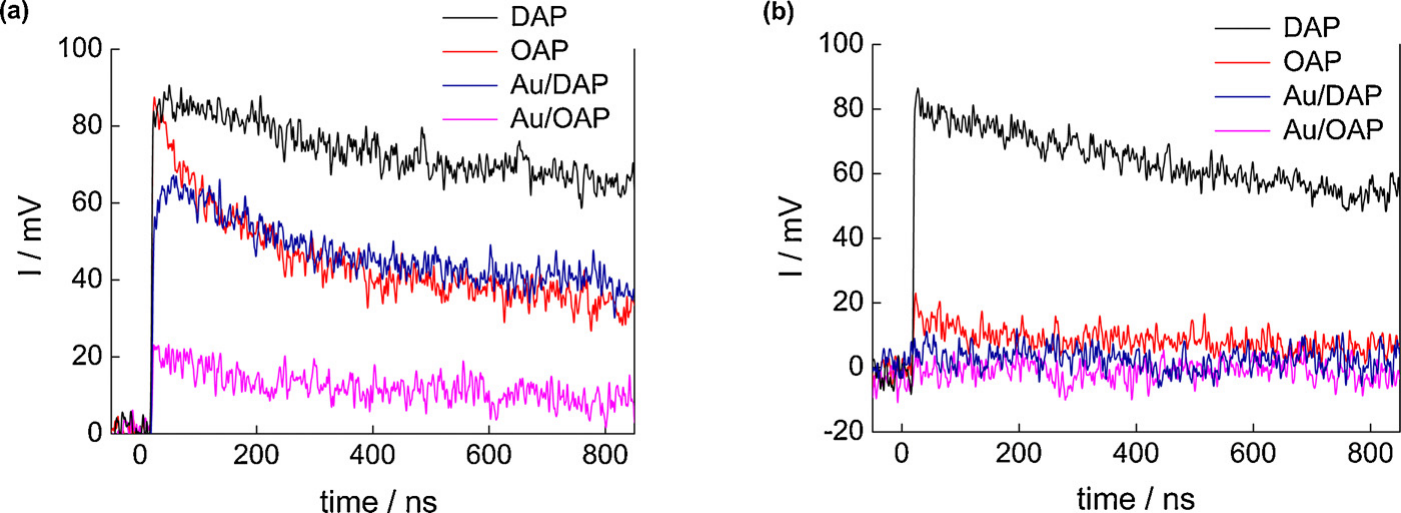
Time course of TRMC signal for bare and Au-modified OAP and DAP, (a) λ_ex_ = 355 nm, (b) λ_ex_ = 545 nm.

For acetic acid decomposition under UV–vis irradiation, bare DAP shows slightly higher activity than bare OAP (Figs. 11b and S6). It should be pointed that both faceted titania samples possess one of the highest photocatalytic activity among titania photocatalysts (like that of titania P25). It is proposed that high photocatalytic activity of OAP is caused by: (i) high content of shallow ET responsible for fast electron transfer (instead of deep ET with permanent electron trapping) [88], as shown in Fig. 4, Fig. 14c, and (ii) preferential adsorption and reduction of oxygen on {101} facets [129]. Whereas, the co-presence of two kinds of facets resulting in an efficient separation of charge carriers (reverse charge transfer: electrons to {101} and holes to {001}, Fig. 14a) has been proposed as the main reason of high photocatalytic activity of bare DAP [130,131]. TRMC analysis confirms an inhibition of charge carriers’ recombination on DAP (Fig. 12a; incredibly slow decay of TRMC signal). Photocatalytic activity significantly increased after titania modification since metal NPs scavenged the photogenerated electrons inhibiting their recombination with holes, as confirmed by a decrease in maximum of TRMC signal. All DAP samples have higher photocatalytic activity than that of respective OAP samples. Modification with gold resulted in larger activity improvement for DAP than for OAP (2.1 v. 1.7 times), and the activity of Au/DAP was ca. 2 times higher than that of Au/OAP. The direct oxidation of acetic acid by positive holes could proceed on {001} facets with a high density of surface undercoordinated Ti atoms (and thus higher reactivity for dissociative adsorption of reactant molecules) because gold and oxygen are preferentially deposited/adsorbed on {101} facets.

**Fig. 13:**
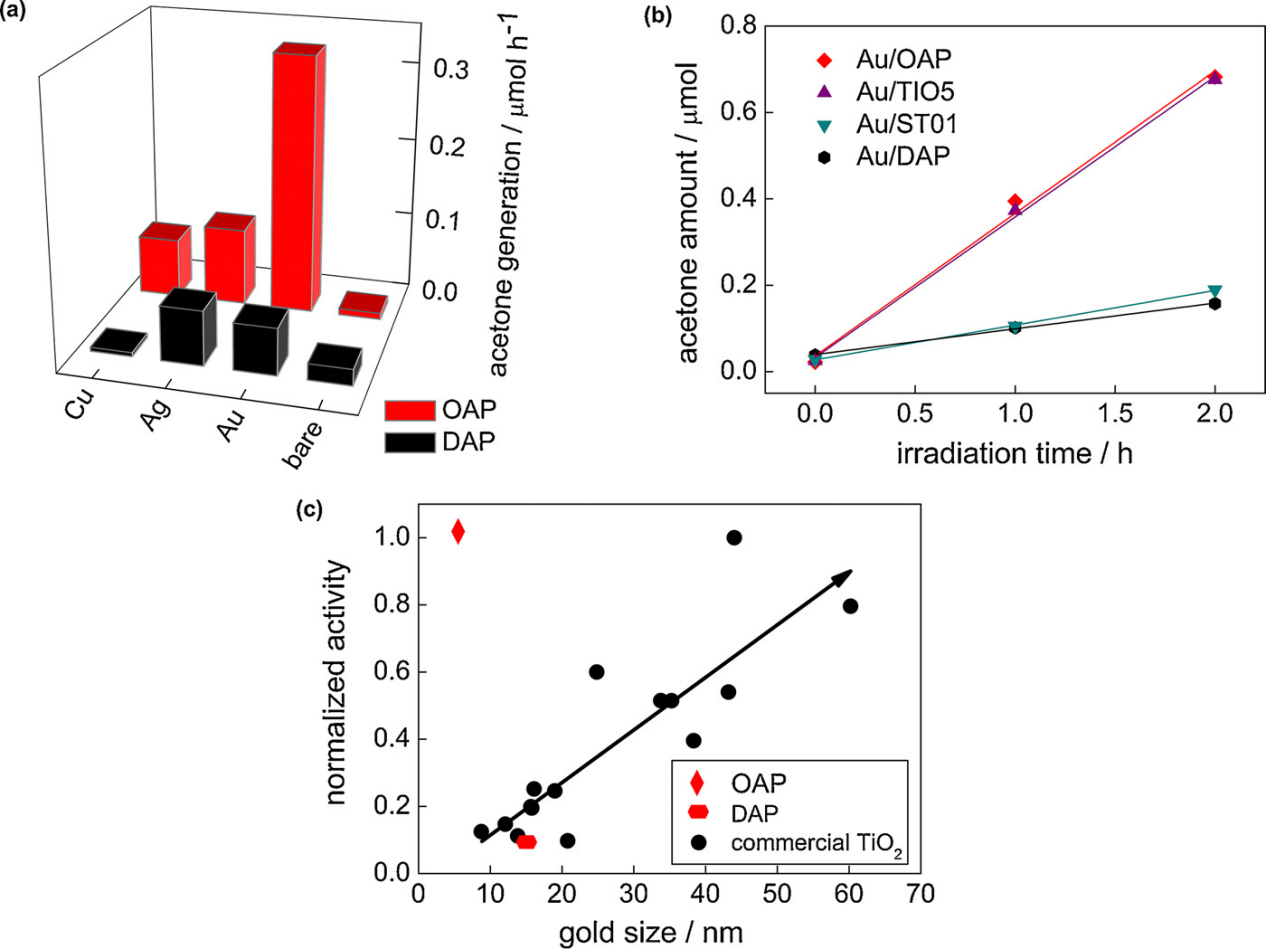
(a) Visible light-photocatalytic activity for 2-propanol oxidation on bare and metal-modified OAP and DAP. (b) Comparison of visible-light photocatalytic activity of gold-modified samples based on faceted and commercial titania. (c) Correlation between gold particle size and photocatalytic activity of OAP, DAP and commercial TiO_2_.

**Fig. 14:**
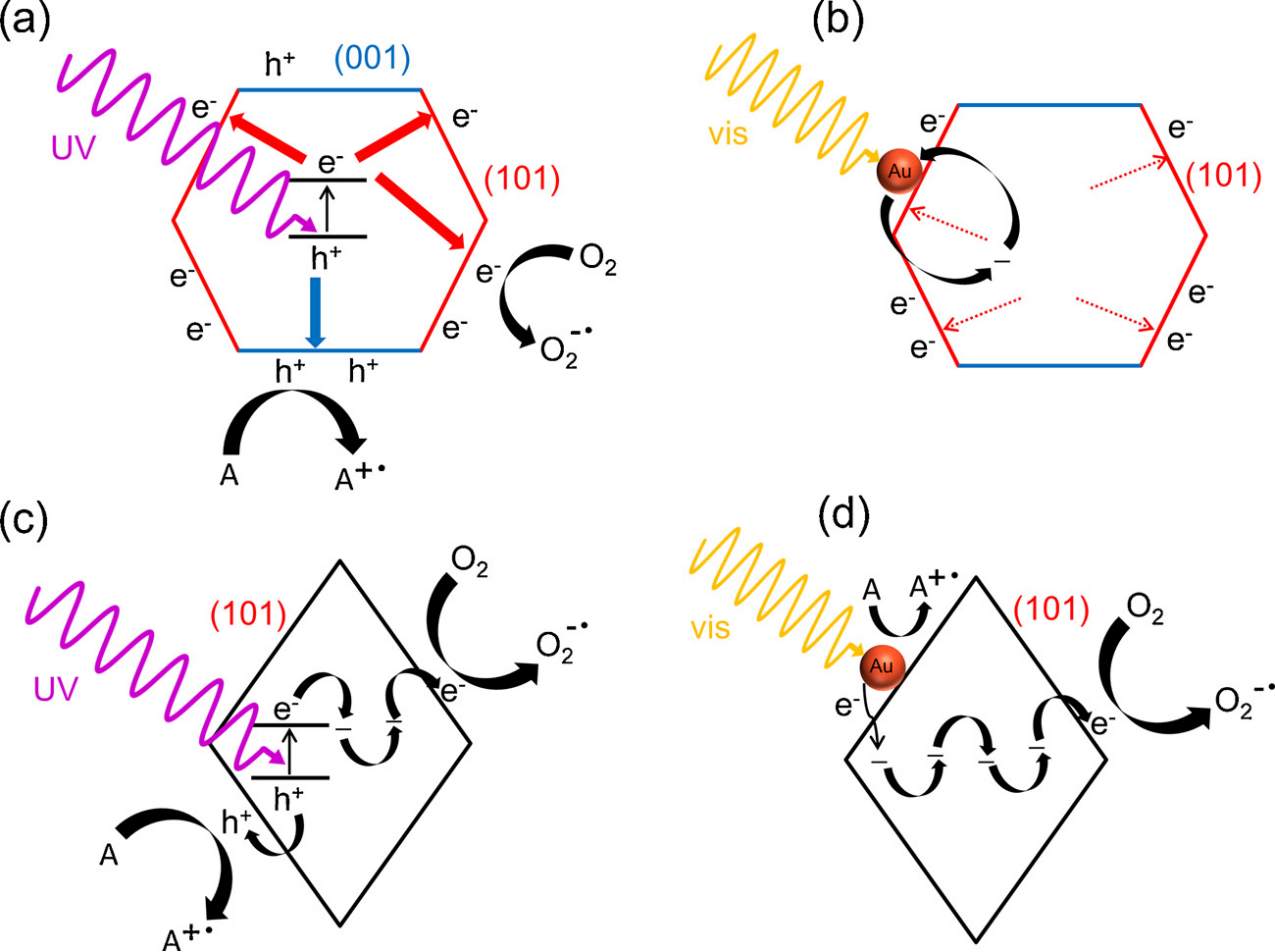
Decahedron (a,b) versus octahedron (c,d) – different mechanisms of photocatalytic reactions under UV (left) and vis (right) irradiation.

Although titania is inactive under vis irradiation, bare DAP exhibits slight activity (λ > 420 nm, Figs. 13a and S7), because of the presence of ET inside bandgap (Fig. 4 and Table 5 (Ti^3+^)). This activity disappears under irradiation with longer wavelengths (λ > 450 nm), although mobile electrons are still detected for DAP even at excitation with 545 nm (Fig. 12b; possibly due to deep ET of energy too low for one- and two-electron reduction of oxygen). In contrast to activities under UV irradiation, all modified OAP samples showed higher photocatalytic activity than that of respective DAP samples. In the case of copper, the photocatalytic activity of Cu/OAP exceeds that of Cu/DAP more than one order of magnitude. The vis response of Cu-modified titania can result from (i) LSPR of copper, (ii) excitation of copper oxides with narrower bandgaps than that of titania (1.3–1.6 eV for CuO and 2.0–2.2 eV for Cu_2_O [132]; as confirmed by reference experiments (Fig. S9), (iii) the interfacial charge transfer from the valence band of titania to the Cu_x_O clusters (x = 1–2) [117]. Considering photoabsorption properties of Au- and Ag-modified samples, DAP samples should exhibit higher photocatalytic activity than OAP samples, due to much broader LSPR peaks (resulting from polydispersity of metal NPs). Surprisingly, much higher activity was obtained for Au/OAP with very fine and uniform gold NPs. To clarify the decisive factor for activity, gold-modified faceted titania samples were compared to Au/TiO_2_ photocatalysts prepared from commercial titania samples (Fig. 13b,c) [96]. It was found that Au/OAP showed the highest photocatalytic activity among fifteen Au/TiO_2_ samples. The high activity of Au/OAP (despite poor photoabsorption properties) and low photocatalytic activity of Au/DAP (noncorrelating with gold-size dependence activity (Fig. 13c)) indicate that titania morphology is one of the key-factors for vis response. Consequently, it is proposed that the presence of {101} facets governs high photocatalytic activity, i.e., due to preferential distribution of shallow than deep ET, and thus high electron mobility (“hot” electrons transfer from Au via shallow ET to adsorbed oxygen on {101} facet, as shown in Fig. 14d). In contrary, very low photocatalytic activity of Au/DAP (with also {101} facets; ca. 80%) suggests that co-presence of {001} facets inhibits activity under vis irradiation. This is very surprising since this facet of high energy is known from its high activity in many different reaction systems.

The contrary results on the influence of titania size on photocatalytic activity were reported, i.e., an increase in photocatalytic activity with: (i) an increase in titania size due to delay of back electron transfer to gold NPs [133], (ii) a decrease in titania thickness (around gold(core)-silver(shell)) due to decrease in probability of trapping electrons before their arrival at the titania surface [134]. The present study could confirm the latter since small OAP modified with NMs exhibits higher photocatalytic activity than modified DAP. It is worth to notice that in the case of Ag NPs deposited also on {001} facets the preferential transfer of electrons from {001} facet to {101} facet [129] could increase the probability of electron permanent trapping. However, the total content of ET in considered faceted titania samples (especially in DAP) is very low (19 μmol g^−1^ (DAP) and 34 μmol g^−1^ (OAP)) comparing to other titania samples [99], and very slow decay of TRMC signal indicate low probability of electron permanent trapping in those faceted titania samples.

It should be pointed that fast recombination of “hot” electrons with holes (or their capture in electron traps) is the main reason of low quantum yields of plasmonic photocatalysts. Therefore, recent studies have focused on inhibition of this recombination, e.g., by preparation of special nanostructures allowing efficient transfer of hot electrons. For example, in the case of titania mesocrystals, deposition of gold NPs on basal surface of titania network (instead of lateral one) resulted in significant enhancement of photocatalytic activity due to efficient electron transfer via titania network [87]. Consequently, it is proposed that the transfer of electrons from {101} facet (on which Au is deposited) against intrinsic properties of decahedral crystal (transfer of electrons to {101} and holes to {001}) could be the main reason of this low photocatalytic activity of modified DAP samples (fast recombination: Au → {101} → Au), as shown in Fig. 14b. Accordingly, it is proposed that deposition of gold on {001} facet should result in enhanced photocatalytic activity (Au_{001}_ → {101} → O_2_), and this has been confirmed by preliminary experiments where deposition of gold NPs on both facets resulted in ca. 60% increase of photocatalytic activity. The present study indirectly supports the mechanism of electron transfer rather than energy transfer since for energy transfer the stronger light absorption rather than the direct contact between metal NPs and titania is crucial.

**Fig. 15:**
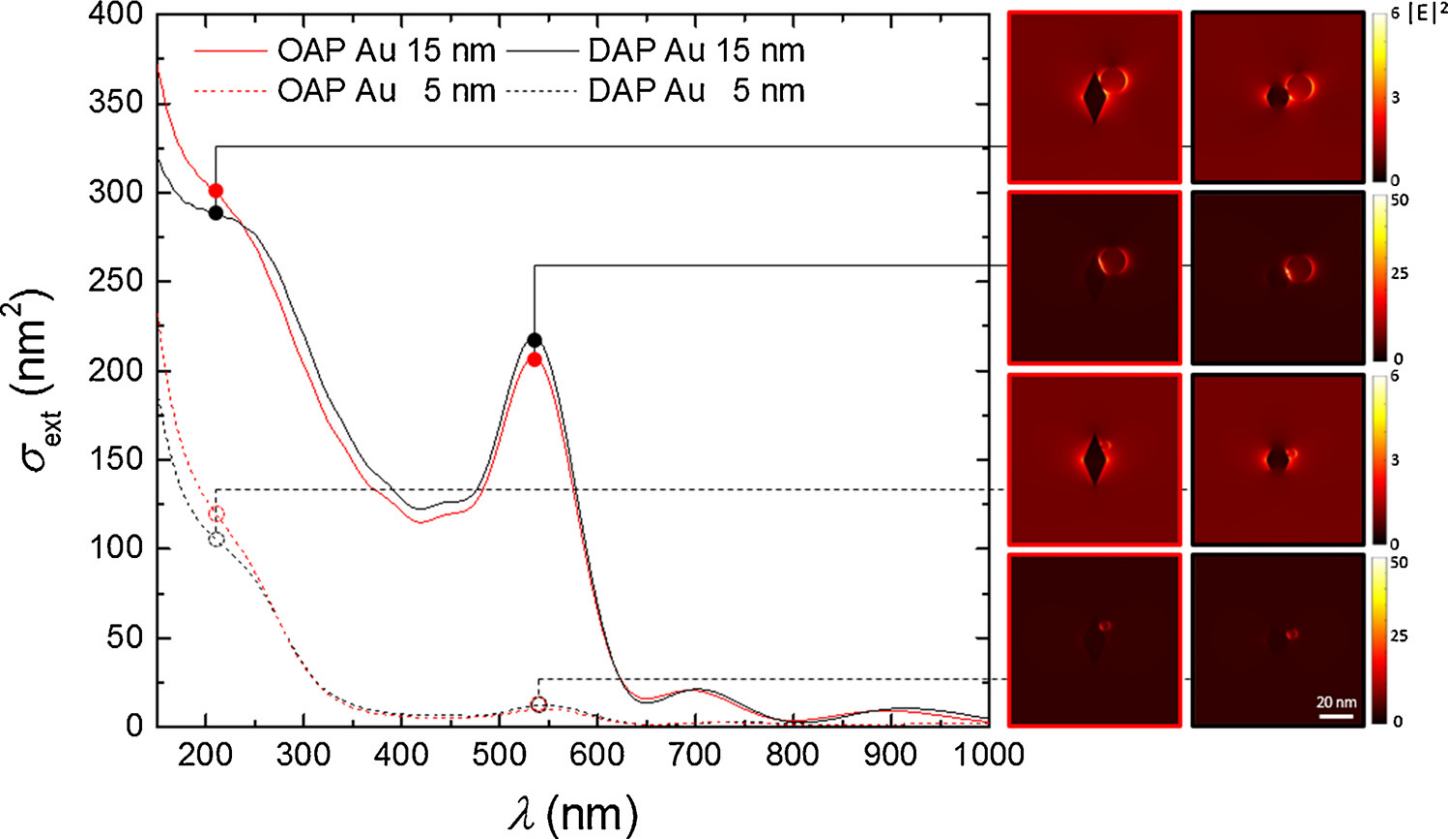
Simulated optical extinction spectra of OAP and DAP nanoparticles with 15 and 5 nm gold nanospheres. Right-side insets show light intensity E^2^ for the plasmonic resonance and at strong UV wing of spectrum.

The discussion on mechanism and determination of key-factors of activities should assume that both photocatalysts do not differ significantly by photoabsorption properties. Although, broader LSPR was observed for Au/DAP than for Au/OAP, the position of LSPR peaks did not differ significantly for both samples. To investigate photoabsorption properties numerical simulation (using finite-difference time-domain (FDTD) method (Lumerical)) of light field enhancement was performed, and obtained spectra of the extinction cross section σ_ext_, which define total losses due to absorption and scattering, for both faceted anatase samples with gold NP of 5 nm and 15 nm are shown in Fig. 15. Scattering is negligible and the extinction corresponds to absorption by gold at these nanoparticle sizes (larger NPs than 100 nm scatter/reflect more than absorb). The insets show the light intensity distribution at the plasmonic resonance and at a UV shoulder of the spectrum. An order of magnitude light enhancement is created at the vicinity of the gold contact with titania and is recognizable by the dipole feature. It should be pointed out that spectra for both titania samples (OAP and DAP) do not differ significantly. At 400 nm wavelengths where titania absorbs light, there is a smaller extinction (absorption) as compared with plasmonic resonance. Due to a high 2.6 refractive index of titania around the plasmonic peak, the magnetic dipole resonance can be excited that is localized inside the titania nanoparticle. Such magnetic dipole provides a stronger light enhancement than the dipole resonance and is titania nanoparticle size dependent. Absorption of light inside titania can facilitate charge separation (Fig. 14) and calls for further investigation of titania-gold nanoparticle systems.

Therefore, the similar light enhancement around OAP and DAP decorated with Au NPs (insets in Fig. 15) indicates that titania shape does not influence significantly the plasmonic properties of NMNPs. In addition, much stronger photoabsorption properties for larger gold NPs (15 nm than 5 nm; Fig. 15, and also Fig. 3) suggest that Au/DAP should exhibit much higher photocatalytic activity than Au/OAP. Therefore, opposite results of photocatalytic activity (than that suggested from plasmonic field simulations) indicate that not physical properties (plasmon field enhancement) but chemical reaction/mechanism (charge carriers’ transfer inside faceted NP) is responsible for higher activity of Au/OAP than that of Au/DAP.

## 4 Summary and conclusions

In conclusion, it has been shown that the modification of OAP and DAP by NMs enhances their photocatalytic performance. The direction of improvement relates to the type of photodeposited metal, faceted titania morphology and selected reaction system. The presence of only {101} facets for OAP or both {101} and {001} facets for DAP – plays a key role in understanding the mechanism of photocatalytic reactions, especially the role of metals in the system. Considering UV–vis reaction systems, two faceted-configuration of DAP is more favorable for the reaction efficiency than single-faceted OAP because of an efficient charge separation which means the transfer of electrons to {101} facets and holes to {001} facets. In the case of vis-induced reactions, metal-modified OAP samples possess higher activity than respective DAP samples. Although, it could be concluded that the presence of {001} facets is detrimental for vis photocatalytic activity, induced by plasmonic mechanism (unlike one-faceted OAP), it is proposed that the localization of NMNPs on DAP is decisive. Therefore, selective deposition of gold/silver NPs on {001} facets of DAP should result in significant improvement of photocatalytic activity under vis irradiation, and thus high overall activity under solar radiation enabling its commercial applications.
