# Supplementary material for: Noble metal-modified faceted anatase titania photocatalysts: Octahedron versus decahedron
Source: Appl Catal B. 2018 Dec 5;237:574–87. doi: 10.1016/j.apcatb.2018.06.027 (PMC6100264; doi:10.1016/j.apcatb.2018.06.027)
Supplement: Supplementary file 1 [file mmc1.pdf]

Supporting Information for

Noble Metal-Modified Faceted Anatase Titania Photocatalysts: Octahedron versus Decahedron

Zhishun Wei,[a,b] Marcin Janczarek ,[b,c] Maya Endo,[b] Kunlei Wang,[b] Armandas Balčytis,[d] Akio Nitta,[b] Maria G. Méndez-Medrano,[e] Christophe Colbeau-Justin,[e] Saulius Juodkazis,[d] Bunsho Ohtani,[b] Ewa Kowalska[b,]*

[a] *Hubei Provincial Key Laboratory of Green Materials for Light Industry, Hubei University of Technology, Wuhan 430068, China*
[b] *Institute for Catalysis, Hokkaido University, N21, W8, 001-0021 Sapporo, Japan*
[c] *Department of Chemical Technology, Gdansk University of Technology, G. Narutowicza 11/12, 80-233 Gdansk, Poland*
[d] *Center for Micro-Photonics, Swinburne University of Technology, John St., Hawthorn 3122 Vic, Australia*
[e] *Laboratory of Physical Chemistry, UMR 8000, University of Paris-Saclay, 91405 Orsay, France*

* Corresponding author, Tel.: 81-11-706-9130; fax: 81-11-706-9133.

E-mail address: kowalska@cat.hokudai.ac.jp

Figures and Tables

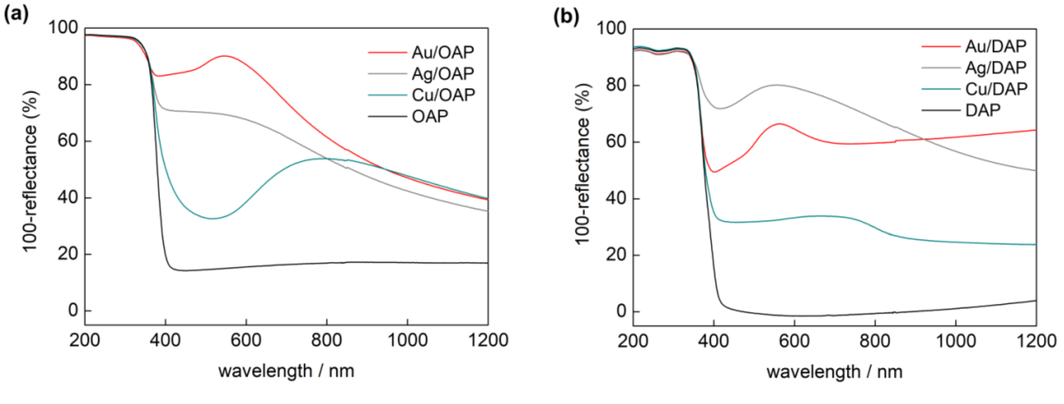

Figure S1. DRS spectra of bare and modified OAP (a) and DAP (b) samples taken with $BaSO_4$ as reference.

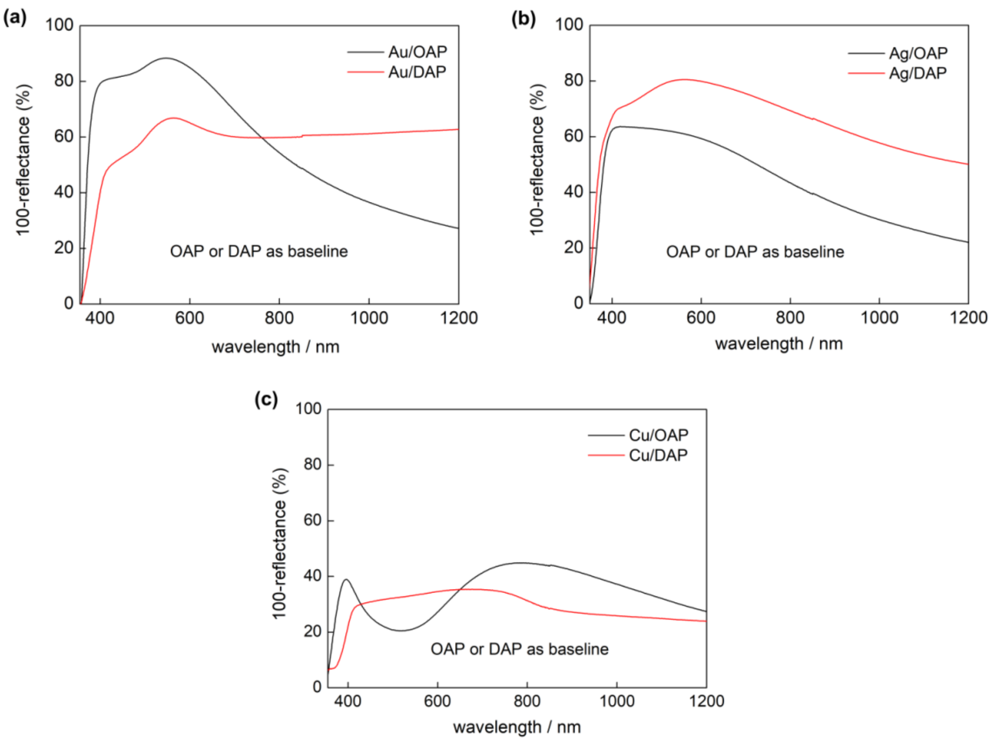

Figure S2. DRS spectra of facetted titania (OAP and DAP) modified with (a) gold, (b) silver and (c) copper taken with bare facetted titania as reference.

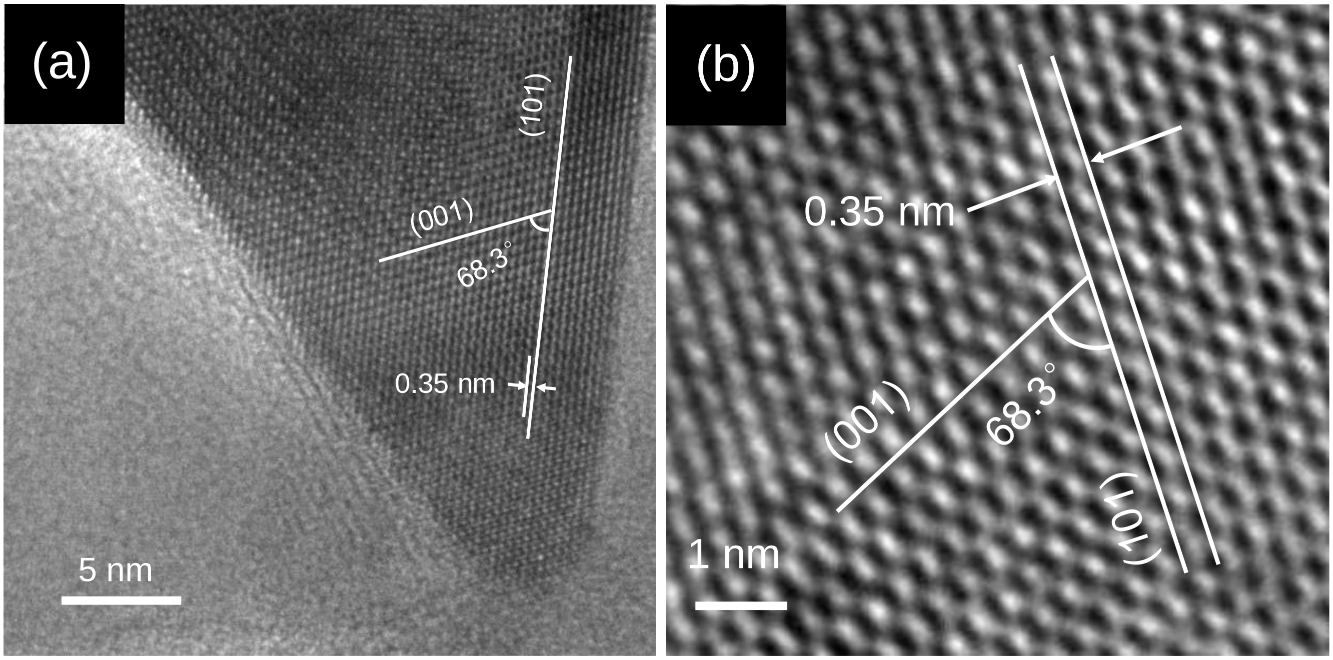

Figure S3. HR-TEM images of OAP for lower (a) and higher (b) resolution modes (0.35-nm lattice distance between fringes and 68.3° angle between {001} and {101} facets correspond to single anatase crystals).

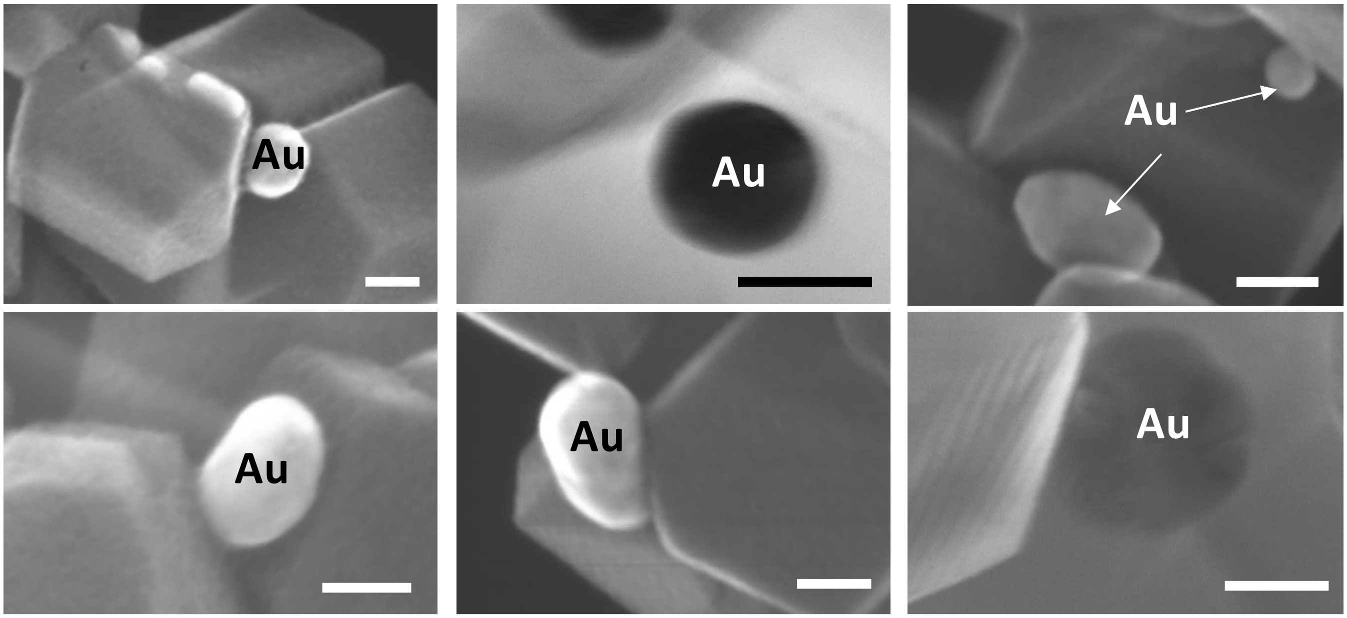

Figure S4. STEM images of Au/DAP (All scale bars correspond to 20 nm).

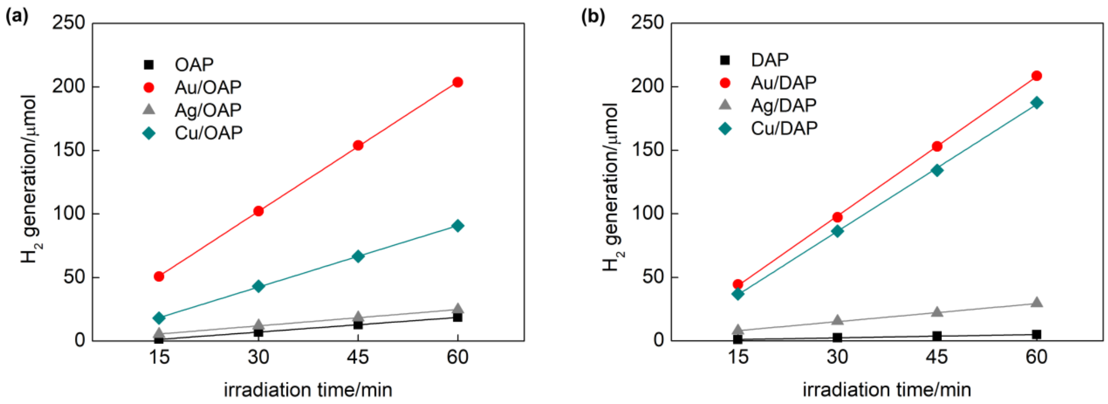

Figure S5. Photocatalytic activity for methanol dehydrogenation on bare and metal-modified OAP (a) and DAP (b).

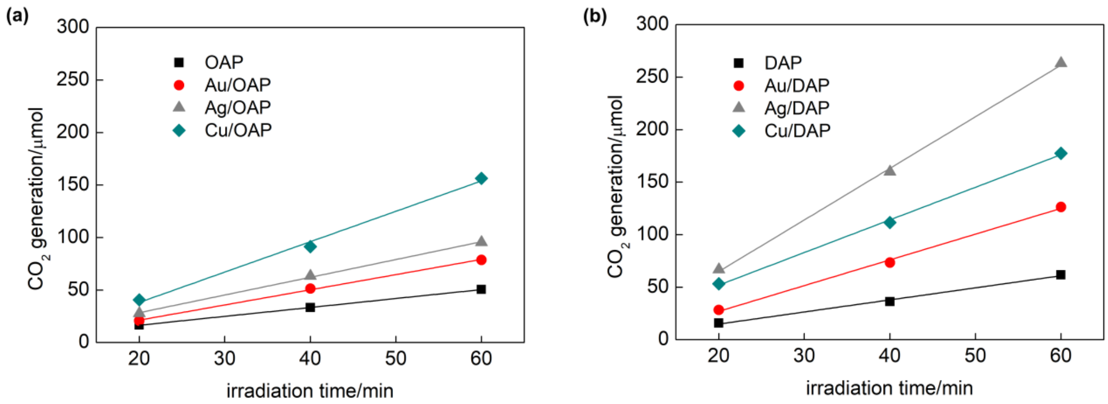

Figure S6. Photocatalytic activity for decomposition of acetic acid on bare and metal-modified OAP (a) and DAP (b).

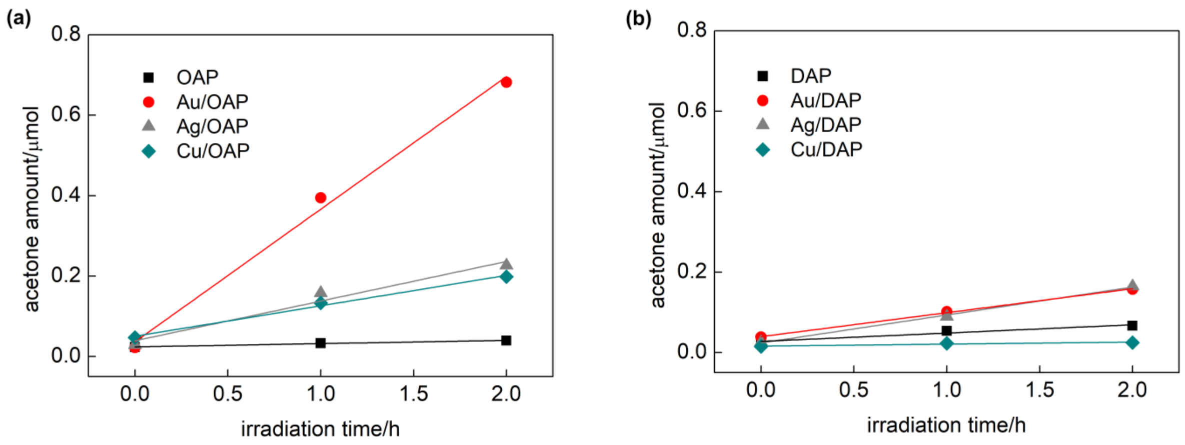

Figure S7. Photocatalytic activity for oxidation of 2-propanol on bare and metal-modified OAP (a) and DAP (b).

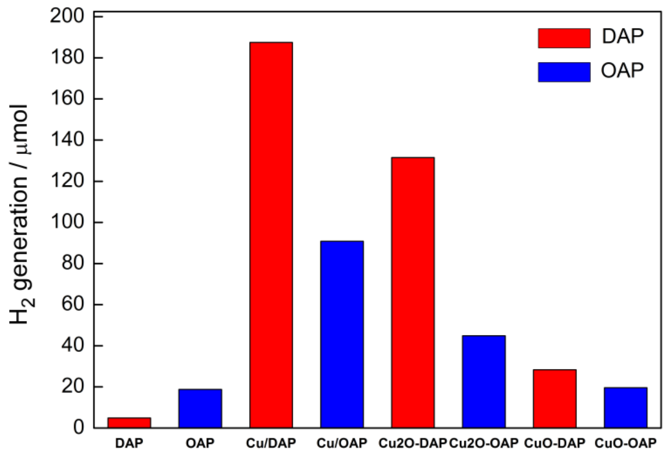

Figure S8. Comparison of photocatalytic activity for methanol dehydrogenation on bare, Cu/OAP, Cu/DAP and physical mixtures of copper oxides (Cu_2O and CuO) and titania (Cu_2O-OAP, CuO-OAP, Cu_2O-DAP and CuO-DAP) under UV/vis irradiation.

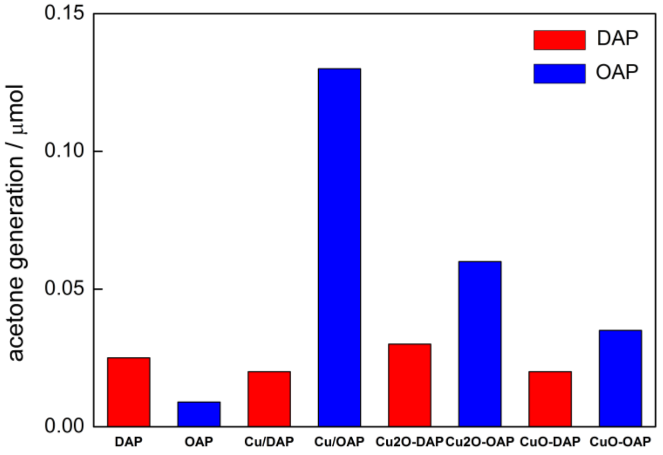

Figure S9. Comparison of photocatalytic activity for oxidation of 2-propanol on bare, Cu/OAP, Cu/DAP and physical mixtures of copper oxides (Cu_2O and CuO) and titania (Cu_2O-OAP, CuO-OAP, Cu_2O-DAP and CuO-DAP) under vis irradiation.
